# Vitamin K and D Supplementation and Bone Health in Chronic Kidney Disease—Apart or Together?

**DOI:** 10.3390/nu13030809

**Published:** 2021-03-01

**Authors:** Marta Ziemińska, Beata Sieklucka, Krystyna Pawlak

**Affiliations:** 1 Department of Monitored Pharmacotherapy, Medical University of Bialystok, 15-222 Bialystok, Poland; marta.zieminska@umb.edu.pl; 2 Department of Pharmacodynamics, Medical University of Bialystok, 15-222 Bialystok, Poland; beata.sieklucka@umb.edu.pl

**Keywords:** vitamin K, vitamin D, chronic kidney disease, bone remodeling, vitamin K and D supplementation

## Abstract

Vitamin K (VK) and vitamin D (VD) deficiency/insufficiency is a common feature of chronic kidney disease (CKD), leading to impaired bone quality and a higher risk of fractures. CKD patients, with disturbances in VK and VD metabolism, do not have sufficient levels of these vitamins for maintaining normal bone formation and mineralization. So far, there has been no consensus on what serum VK and VD levels can be considered sufficient in this particular population. Moreover, there are no clear guidelines how supplementation of these vitamins should be carried out in the course of CKD. Based on the existing results of preclinical studies and clinical evidence, this review intends to discuss the effect of VK and VD on bone remodeling in CKD. Although the mechanisms of action and the effects of these vitamins on bone are distinct, we try to find evidence for synergy between them in relation to bone metabolism, to answer the question of whether combined supplementation of VK and VD will be more beneficial for bone health in the CKD population than administering each of these vitamins separately.

## 1. Introduction

Chronic kidney disease (CKD) represents a global health issue involving about 13% of the general population, of which about 11% are patients in the 3–5 stage of CKD [1]. Impaired kidney function impacts the quality of bone tissue and results in the development of disorders in bone and mineral metabolism, which are defined as Chronic Kidney Disease-Mineral Bone Disorders (CKD-MBD) [2]. Abnormalities in mineral and bone metabolism contribute, in part, to severity of vascular calcification (VC). In this context, CKD-specific risk factors are believed to drive substantially to VC and cardiovascular disease. It is also established that patients with CKD stage 3–5 will die due to cardiovascular events before the need of renal replacement therapy [3,4].

**Figure 1:**
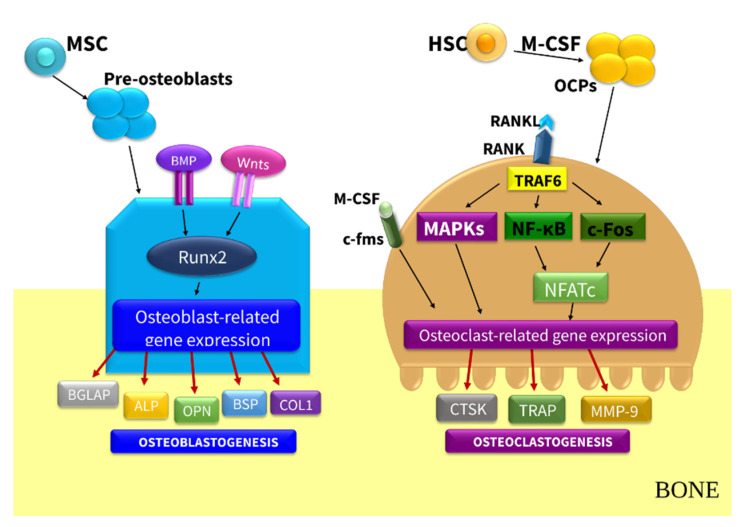
Bone remodeling. Abbreviations: ALP, alkaline phosphatase; BGLAP, bone-Gla-protein; BMP, bone morphogenic protein; BSP, bone sialoprotein; c-fms, colony-stimulating factor-1 receptor; COL1, collagen type 1; CTSK, cathepsin K; HSC, hemapoietic stem cells; MAPKs, mitogen-activated protein kinases; M-CSF, macrophage colony-stimulating factor; MMP-9, matric metalloproteinase 9; Msc, mesenchymal stem cell; NFATc, nuclear factor of activated T-cells; NFkB, nuclear factor-kappa B; OPN, osteopontin; OCPs, osteoclast precursors; RANK, receptor activator of nuclear factor kappa B; RANKL, receptor activator of nuclear factor kappa B ligand; Runx2, runt-related transcription factor 2; TRAF6, Tumor necrosis factor receptor associated factor 6; TRAP, tartare-resistant acid phosphatase.

CKD-MBD impacts bone remodeling (Figure 1)—the dynamic process mediated mainly by the two antagonistically acting cellular populations: osteoblasts (OBs) that control the formation of bone and osteoclasts (OCs), with the ability to resorb mineralized bone [5]. This process is tightly regulated by local and systemic hormones, such as parathyroid hormone (PTH), 1,25-dihydroxyvitamin D (1,25D), and vitamin K (VK) [5,6]. The process of bone remodeling is composed of four phases: the activation phase (the recruitment of OCs); the resorption phase (the resorption of bone by OCs); the reversal phase (the apoptosis of OCs and the recruitment of OBs); and the formation phase (the OBs lay down new organic bone matrix that subsequently mineralize) [6]. Bone remodeling together with bone size, geometry, structure, and volume determines bone’s biomechanical properties, integrity, and strength, providing renewal of damaged bone. An imbalance between the amount of resorbed bone and the quantity of new bone formation substantially contributes to the increased risk of fractures, which is associated with higher mortality in patients with CKD [7,8,9].

Many clinical studies reported on VK and vitamin D (VD) deficiency in patients with CKD or undergoing dialysis [3,10,11,12]. These vitamin deficiencies could result from both dietary and nondietary determinants. Dietary recommendations for CKD patients, such as diets low in potassium (fewer leafy green vegetables rich in vitamin K1, VK1) and low in phosphate (fewer dairy products rich in vitamin K2, VK2) could promote VK deficiency. Holden et al. [3] showed that patients with stage 3–5 CKD have higher VK1 levels than those on maintenance dialysis. They concluded that patients who were clinically better nourished have also better vitamin K status. Nutritional factors may also affect the deficiency of VD status in CKD. The low food intake was frequently noticed in this population, due to numerous reasons, such as reduced appetite, dietary restrictions, i.e., low protein and phosphate diets, uremic-related gastrointestinal symptoms, and impaired gastrointestinal absorption of VD [13]. The nondietary determinants of VD in a cohort of patients with CKD included age, gender, low physical activity, less sunlight exposure, blunted the response of plasma VD to ultraviolet (UVB) irradiation, and hyperpigmentation, which may play a role in the impaired endogenous VD synthesis [14]. Additionally, with an increased loss of renal tissue, the availability and functionality of 1-α hydroxylase decreases, thereby reducing 1,25D [15]. Proteinuria has also been described as a contributing factor in the pathogenesis of VD deficiency [3,13,14]. Vitamin D binding protein (VDBP) carries about 85% of the circulating 25-hydroxyvitamin D (25D), VDBP–25D complexes are filtered in the glomerulus. Patients with proteinuria usually present the increased urinary VDBP excretion, but they might also show impaired megalin and cubilin-mediated protein reuptake in the proximal tubules, which may contribute to VD deficiency in the setting of CKD and proteinuria, especially in diabetic chronic kidney disease (DCKD) [16,17]. The peritoneally dialyzed patients are at particularly high risk of VD deficiency due to increased loss of 25D and VDBP through the peritoneal effluent [18]. Moreover, the chronic inflammatory state, which is a common feature of CKD, can affect VD status [3].

Nondietary determinants of VK status in CKD include taking drugs prescribed to patients with CKD, such as warfarin, statins, proton-pump inhibitors, phosphate binders, steroids, or antihypertensives drugs [12,19,20]. The genetic variability can contribute to the large interindividual variation in VK biomarkers. Holden et al. [3] showed that Apolipoprotein E4 (ApoE4) carriers may be at risk for undercarboxylated VK-dependent proteins (VKDPs) due to rapid clearance of VK1 in the liver. Thus, the apoE4 allele, carried by 34% of this CKD population, may potentially represent a nonmodifiable risk factor influencing VK status.

Regarding the general population, a recent report recommended that serum 25D concentrations should be maintained at 20–50 ng/mL, and values >30 ng/mL should be considered normal [21]. However, there is some doubt as to whether the values considered “normal” in the general population could be applied to CKD patients. The Kidney Disease Improvement Global Outcomes (KDIGO) guidelines, published in 2017, do not consider any reference value for 25D level in CKD, but they recommended its evaluation when PTH progressively increases or stays above normal at stages of CKD above 3 [2]. A more recent study performed on stage 1–5 CKD patients showed no evidence of a decreasing effect of 25D on PTH lowering until 25D levels of 42–48 ng/mL [22], suggesting a higher VD target in CKD without any additional risk of hypercalcemia and hyperphosphatemia.

Establishment of the reference value of VK in patients with CKD is a challenge, because there is no gold standard for the measurement of VK levels and there is a lack of standardization. Instead, a functional deficiency of VK is commonly used as a surrogate of VK status in these patients. Measurements of uncarboxylated prothrombin (known as protein induced by VK absence/antagonism II (PIVKA-II), uncarboxylated OC (ucOC), and desphospho-uncarboxylated matrix Gla protein (dp-ucMGP) MGP are indicative of VK deficiency [3,11,23].

This review focuses on the contribution of VD and VK to skeletal health in CKD, discussing their effects on bone remodeling, derived from in vitro, in vivo, and clinical studies. In particular, we tried to find a functional synergy between these vitamins in relation to bone health in CKD and answer the question of whether simultaneous supplementation with VD and VK may be more beneficial in counteracting the effects of CKD-MBD than supplementing the deficiency of a particular vitamin.

## 2. Vitamin D and Vitamin K in CKD

### 2.1. General Characteristics of Vitamin K—Chemical Structure, Metabolism, and Laboratory Evaluation

**Figure 2:**
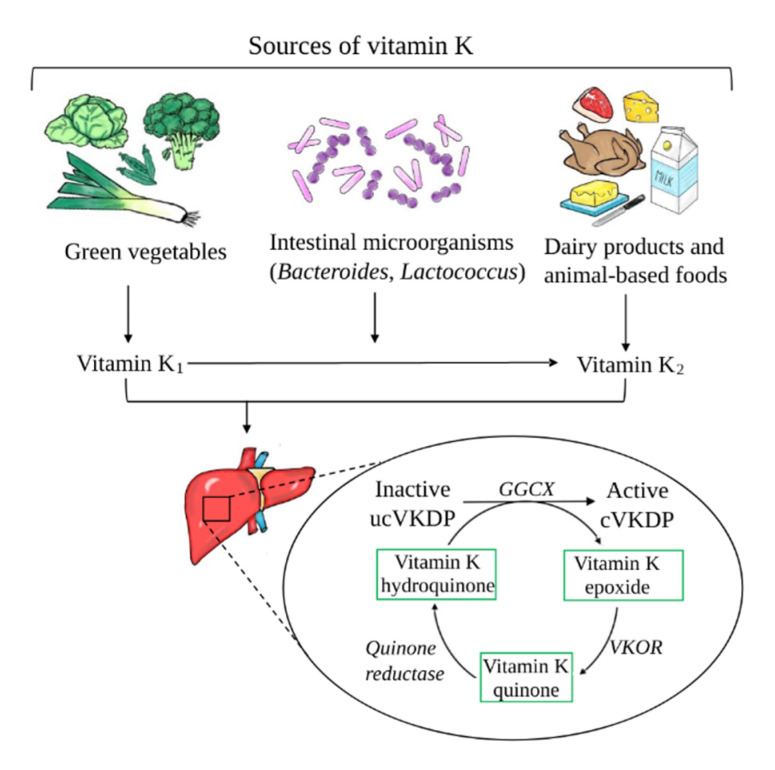
Vitamin K sources, metabolism, and mechanism of action. Abbreviations: ucVKDP, uncarboxylated vitamin K-dependent proteins; cVKDP, carboxylated vitamin K-dependent proteins; GGCX, enzyme γ-glutamyl carboxylase; VKOR, vitamin K epoxide reductase.

VK constitutes a group of fat-soluble chemical compounds, whose common property is a structure containing 2-methyl-1,4-naphthoquinone. Naturally, VK occurs in two forms—VK1 (phylloquinone) and VK2 (including different menaquinones, MKs). VK1 is the main source of dietary VK and is mainly found in green leafy vegetables and plant oils. MKs are derived from intestinal bacteria (*Lactococcus* or *Bacteroides*) and fermented food [24]. The most common MKs in humans are the short-chain MK-4; it is the only MK produced by systemic conversion of phylloquinone to menaquinones. MK-4 can be endogenously produced from phylloquinone in some tissues, which is probably due to local biosynthesis [25]. The recently identified MK-4 biosynthetic enzyme, UbiA prenyltransferase containing 1 (UBIAD1), is widely expressed, but the mechanisms regulating its expression are not currently known [26]. The main sources of VK are illustrated in Figure 2.

The main physiologic role of VK is to act as cofactor for the γ-glutamyl carboxylase (GGCX) enzyme in the gamma-carboxylation reaction that add carboxyl groups to glutamic acid (Glu) residues in proteins. GGCX oxidizes VK into VK epoxide and then adds CO2. The newly carboxylated residues in such proteins are referred to as gamma-carboxyglutamic Gla domains. This process transforms inactive (uncarboxylated) proteins into active carboxylated VKDPs, enabling them to bind to calcium. Adequate calcium binding is a critical physiological step in blood coagulation, bone mineralization, and vascular calcification. The most acknowledged extrahepatic VKDPs are MGP, osteocalcin (OC), growth arrest specific protein 6 (Gas6), and Gla-rich protein (GRP) [27].

In addition to protein modification, a novel mechanism was uncovered in the signaling that regulates the transcription of target genes by VK through the activation of a nuclear receptor, the steroid and xenobiotic receptor (SXR; also known as nuclear receptor subfamily 1 group I member 2 (NR1I2) and pregnane X receptor (PXR), which is the mouse and rat ortholog of SXR) [28]. VK2 was shown to bind to and activate the SXR, which could induce expression of osteoblastic marker genes, such as alkaline phosphatase (ALP) and osteoprotegerin (OPG), extracellular matrix-related genes, and collagen accumulation in osteoblastic cells [29]. When we compare both forms of VK, VK1 is predominant form of VK in the human diet due to its relatively high content in food [30], but VK2 is required for OC to become activated and bind calcium, which makes VK2 a vital player in case of supporting the osteoprotective effect and maintenance of bone health [31,32,33]. Additionally, VK2 plays an important role in promoting bone formation: it stimulates the differentiation of osteoblasts, upregulates the gene expression of bone markers, and inhibits osteoclastogenesis [33]. VK reserves in the body are limited, and it is efficiently recycled through a series of redox reactions, which are defined as the “VK cycle” (Figure 2). The transformation of VK epoxide to quinone form occurs through VK epoxide reductase (VKOR). Then, quinone is converted by quinone reductase to a VK hydroquinone form, which can be reused. This last stage of the VK regeneration cycle is necessary for proper γ-carboxylation, because only a reduced form of VK can act as a cofactor for GGCX [34].

The determination of VK levels is difficult because of its physicochemical properties and low levels of VK in circulation. Measurements can be done using direct and indirect methods. One of the most popular direct methods is the determination of VK using high-performance liquid chromatography (HPLC). A disadvantage of this method is the possibility of interaction between an HPLC column and lipoproteins transporting VK, which may affect the results [35]. The most common indirect method for determining VK status is measuring uncarboxylated VKDPs—ucOC and ucMGP—through the enzyme-linked immunosorbent assay (ELISA). The uncarboxylated forms (uc)VKDP appear when protein carboxylation is decreased and increased levels of ucOC, ucMGP or dp-ucMGP reflect a VK deficiency.

The ucOC level, the total OC level, and the ratio between the two (%ucOC) are frequently used to reflect VK status linked to bone health [36]. The diagnostics of VK deficiency are also based on measuring PIVKA. These proteins are formed in the liver as inactive, under-carboxylated precursors that cannot perform their biological functions. Long-term VK status can be shown by PIVKA II measurement that together with prothrombin time are markers of the hepatic concentration of VK [10].

### 2.2. Vitamin K Status in CKD Patients

Many observational and interventional studies reported that patients with CKD undergoing conservative treatment, peritoneal dialysis (PD), or hemodialysis (HD) suffer from subclinical VK deficiency [3,10,11,20,23,37]. The number of CKD patients with VK deficiency reaches 70%–90% of that population and is more pronounced than in the general population [38]. The alterations in the OC levels have been already observed in the early stages of CKD, as 60%–70% of pre-dialysis patients had a high percentage of serum ucOC [3,39]. Several studies demonstrated that HD patients had poor VK status [3,10,11,20,23,37,40,41], which is rather associated with the dietary regimen and overall poor nutrient intake. In addition to the fact that VK has a lipophilic character, it should not be absorbed or removed by the membrane during dialysis [42,43]. Westenfeld et al. [41] showed that HD patients had significantly higher levels of dp-ucMGP and ucOC as compared to a healthy group, pointing out that most HD patients suffer from a VK deficiency. A recently published study by Cranenburg et al. [23] demonstrated low intake (140 μg/day) of VK1 and VK2 by HD patients. Interestingly, low VK intake was observed on the weekends and days of dialysis in comparison to the control group. Additionally, dp-ucMGP and ucOC were significantly elevated in the majority of HD patients, confirming a subclinical hepatic VK deficiency, whereas high levels of non-carboxylated MGP in these patients pointed to a vascular VK deficiency. Voong et al. [37] showed that the majority of HD patients had high levels of ucOC, and almost 30% had low levels of phylloquinone, confirming a subclinical VK deficiency. A recent observational study by Fusaro et al. [40] showed that total OC and ucOC levels were higher in patients with CKD than in healthy controls.

There are a few studies showing that PD patients have a comparable degree of VK deficiency to HD patients. Stankowiak-Kulpa et al. [44] demonstrated that 46% of PD patients had a VK insufficiency, as measured by elevated PIVKA-II levels. Another cross-sectional study of PD patients [11] showed that almost 30% of them had a VK deficiency, as assessed by serum VK1 level, and all patients had a VK deficiency, as measured by the level of ucOC. Interestingly, Jansz et al. [45] demonstrated that patients after kidney transplantation had lower levels of dp-ucMGP compared to HD or PD patients, indicating that the restoration of kidney function may contribute to an improvement in VK status.

It is widely known that VK is crucial for the activation of OC, which is involved in bone metabolism. OC (also known as bone-Gla-protein, BGLAP) is one of the main noncollagenous proteins that is synthetized by OBs during bone formation. Modification by VK-dependent carboxylation converts ucOC to an active carboxylate form (cOC). The cOC binds calcium ions and incorporates them into hydroxyapatite crystals in the bone matrix to promote bone formation [46]. Transcription and translation of the OC gene is under the control of 1,25D [47] and PTH [48], creating immature ucOC. Circulating OC is used as a good biomarker of bone formation, whereas high ucOC levels are an expression of poor VK levels and intake [43]. However, when bone is resorbed, OC fragments are released into the circulation, and their serum concentrations may reflect bone turnover. In a healthy organism, the proportion of ucOC to total OC typically does not exceed 20% [49]. OC clearance is through glomerular filtration; hence, patients with CKD demonstrate significantly increased levels of total serum OC and ucOC compared with healthy controls [10,23,39,41,50].

### 2.3. General Characteristics of Vitamin D

VD is a prohormone that acts in a variety of paracrine and autocrine systems. VD exerts a pleiotropic effect in the body, plays an important role in calcium–phosphate homeostasis and the regulation of PTH, bone metabolism, immune system, and cardiovascular disease [51,52].

**Figure 3:**
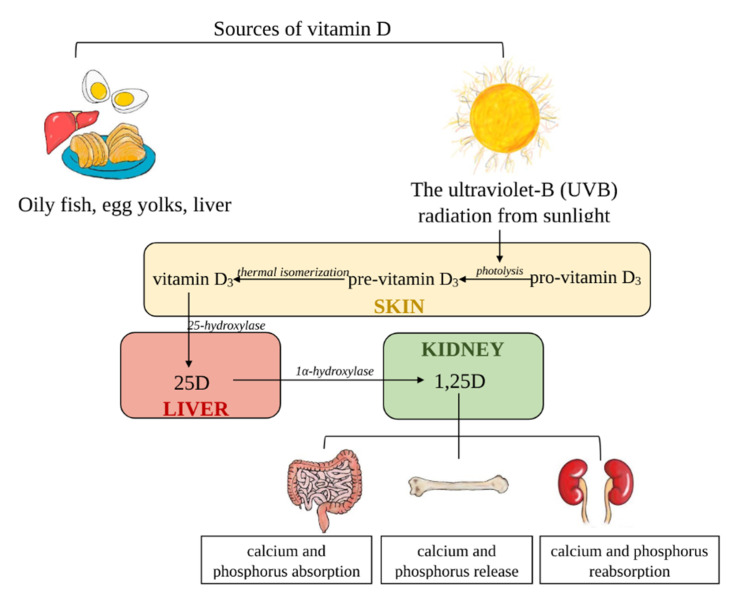
Vitamin D sources, metabolism, and role in calcium homeostasis. Abbreviations: 25D, 25-hydroxycholecalciferol; 1,25D, 1,25-dihydroxycholecalciferol.

VD is a fat-soluble vitamin that exists in two distinct forms, ergocalciferol (VD_2_) and cholecalciferol (VD_3_) [12]. The sources of VD_2_ are vegetables and “fortified” food, whereas VD_3_ is derived from animal-based foods but is mainly synthetized in the skin [12,13]. VD_3_ is produced through the action of ultraviolet (UV) sunlight in the skin by photolytic conversion of 7-dehydrocholesterol (pro-VD_3_) to pre-VD_3_ (precalciferol); then, it is subsequently is changed to VD_3_ [53,54]. Due to the fact that both forms of vitamin D (VD_2_ and VD_3_) are biologically inactive, they need further metabolism to be activated. In the next step, they are transported by VDBP in the liver, where they are subjected to the hydroxylation process by 25-hydroxylase (CYP2R1) to create 25D (calcidiol) [55]. The final step of VD activation, a second hydroxylation, occurs in the kidneys, where 25D is transformed into a biologically active form of 1,25D (calcitriol) by 1α-hydroxylase (CYP27B1) [56]. The level of the circulating form of 25D is 1000-fold higher than the active form of VD—1,25D [54,57,58]. The main sources of VD and their metabolism are illustrated in Figure 3.

In a healthy individual, the kidneys are the main place of 1,25D synthesis, but under specific conditions (CKD, rheumatoid arthritis, pregnancy), other cell types can also release it into circulation [55]. Interestingly, these extrarenal 1,25D products do not include the 1,25D pool [55]. Additionally, renal production of the active form of VD is strictly dependent on substrate availability, when 25D concentration is low [57,58]. A wide range of biological actions are mediated through binding with the VD receptor (VDR) and lead to changes in the expression of many genes e.g., Receptor Activator for Nuclear Factor κ B Ligand (RANKL), Low-density lipoprotein receptor-related protein 5 (LRP5), Cytochrome P450 family 24 subfamily A member 1 (CYP24A1), and Transient Receptor Potential Cation Channel Subfamily V Member 6 (TRPV6) [59,60]. The circulating active forms of VD are highly regulated by many hormones, e.g., PTH, fibroblast growth factor 23 (FGF-23), low blood calcium, or phosphorus concentration [59]. VD and PTH interact in a tightly controlled feedback cycle and play a major role in the regulation of calcium and phosphate homeostasis [61]. VD deficiency with hypocalcemia and decreased calcium absorption from diet leads to enhanced PTH secretion, which results in increased renal calcium reabsorption and osteoclastic bone resorption [62,63,64]. PTH and hypocalcemia enhance CYP27B1 pathway-mediated hydroxylation of 25D to its active form: 1,25D. Therefore, 1,25D augments Ca^2+^ absorption in small intestines, increases PTH-dependent Ca^2+^ reabsorption in kidney, and mediates PTH-stimulated calcium release from bone [62]. Thus, PTH is a pivotal stimulator of VD synthesis, while on the other hand, VD has a negative influence on PTH secretion [61].

Serum 25D concentration is the most reliable biomarker for assessing VD status. To date, there is a lack of standardized methods for quantifying the level of 25D [65]. Although the gold standard for evaluating VD status is HPLC, it is not widely used due to high costs as well as the need for experience and special equipment. The second method that is extensively used to establish the reference range of serum VD is the DiaSorin Liaison assay, which is a quantitative chemiluminescent immunoassay (CLIA) [66,67]. Other common methods include ELISA and radioimmunoassay (RIA) [65,68]. Lately, more attention has been paid to liquid chromatography-tandem mass spectrometry (LC-MS/MS), which is able to measure the various serum forms of VD and VD_3_ [69].

### 2.4. Vitamin D Deficiency in CKD Patients

Low levels of 25D are common in CKD as well as in the general population, but the prevalence of 25D deficiency is much greater in the CKD population [70,71]. VD deficiency/insufficiency affects more than 80% of patients with CKD [14,65]. Moreover, many observational and interventional studies reported that kidney transplant recipients are susceptible to low levels of VD [72]. A VD deficiency increases with the progression of CKD, and it accounts for 20% in CKD stage 3 and 30% in CKD stages 4–5 [73]. Interestingly, Cankaya et al. demonstrated that PD and HD patients’ VD levels were lower in comparison with CKD and renal transplant patients [74]. The low VD status has been related to increased progression of kidney and bone disease [75,76], cardiovascular events, metabolic syndrome, vascular calcification, ventricular hypertrophy, muscle weakness, insulin resistance, and overall mortality in this population [12,75,76].

Both the Kidney Disease Outcomes Quality Initiative (KDOQI) and KDIGO guidelines recommend checking and supplementing low serum 25D levels in CKD and dialysis patients [2,77]. Additionally, KDIGO experts suggest that VD concentrations in patients with CKD should be tested; thus, repeated measurements should be individualized as a result of the baseline values and interventions. However, there is no consensus on how frequently VD level should be measured and administered [2,77].

## 3. Role of Vitamin K and Vitamin D in Bone Remodeling in CKD: Pre-Clinical Evidence

### 3.1. Vitamin K and Bone Remodeling—In Vitro Studies

VK (in particular K2) improves the function of OBs by inducing their proliferation, differentiation [78,79], and inhibiting Fas-mediated OB apoptosis [80]. VK2 treatment of OBs can increase both ALP activity and the level of bone OC in the cell medium [78]. The higher ALP activity is associated with better formation of the organic matrix and the mineral part of the bone, and the deposition of OC and hydroxyapatite in the bone. VK2 activates SXR [28,29] and operates as a transcriptional regulator of a number of osteoblastic biomarker genes and extracellular matrix-related genes [29,81]. Moreover, VK2 supports bone formation and suppresses bone resorption by stimulating the expression of OPG and inhibiting the expression of RANKL on OBs [78].

Yamagushi et al. [82] observed that VK2 induced the downregulation of basal and cytokine-induced nuclear factor kappa-light-chain-enhancer of activated B cells (NF-*κ*B) expression in OBs as well as in the OC precursors, explaining its dual pro-anabolic and anti-catabolic activities. Interestingly, the combined use of VK2 and 1,25D enhanced calcium deposition and OC expression in the OBs of obese diabetic mice [83], suggesting that this combined therapy may be more effective for the treatment of diabetes associated osteoporosis than the use of VK alone. On the other hand, the current evidence suggests that VK2 reduces osteoclastic activity via different strategies. It prevents OC formation either directly or indirectly (by interfering with the RANKL/OPG system [78]). VK decreases both the proliferation of tartrate-resistant acid phosphatase (TRAP) positive cells and TRAP activity in osteogenic culture medium [78,84]. Moreover, VK2 inhibits bone resorption induced by bone resorbing factors, such as Prostaglandin E2 (PGE2), Interleukin 1*α* (IL1*α*), and 1,25D [85]. The study of Kameda et al. [84] showed the potential of VK2 to induce OCs apoptosis. The characteristics of the main studies regarding VK in pre-clinical studies included in the review are shown in Table 1.

### 3.2. Vitamin D and Bone Remodeling—In Vitro Studies

#### 3.2.1. Impact of 1,25D on Osteoblast Function

In the available literature, there are few data on the in vitro effect of 1,25D on OBs from patients with CKD. Table 1 summarizes the main pre-clinical studies regarding VD. The first report by Zhou et al. [91] showed that different forms of VD, 25D and 1,25D, can stimulate in vitro OBs differentiation of marrow stromal cells from healthy controls and CKD subjects. Several years later, it was shown that primary OBs derived from CKD patients display a maturation defect in vitro [92]. A recent study of this team [86] documented that 1,25D markedly stimulated the expression of FGF-23, and the mature OB marker, BGLAP, in primary OBs derived from CKD patients. However, recombinant human FGF-23 countered VD-stimulated OBs differentiation of human bone marrow stromal cells (hMSCs) by reducing VDR and CYP27B1 expression as well as inhibiting 1,25D biosynthesis and signaling through bone morphogenic protein-7 (BMP-7) [93]. 1,25D in very high concentration (100 nM), which far exceeds the concentrations achieved in dialysis patients receiving high doses of calcitriol, improved in vitro OBs mineralization. On the other hand, VD stimulated the expression of the osteoclast differentiation factor, RANKL, in primary CKD OBs, and especially its high doses (10 nM and 100 nM) increased the ratio of RANKL/OPG expression. In contrast, VD sterols had no effect on the expression of the early osteoblastic marker, Runt-related transcription factor 2 (RUNX2), and they had very little effect on ALP expression in CKD cultures. These data suggest that 1,25D may play an important role in OBs maturation by regulating osteoclast–osteoblast coupling in the bone of CKD patients [86].

Over the course of the last decades, 1,25D has been studied extensively for its pleiotropic actions promoting bone remodeling in the general population, and numerous in vitro studies have implicated 1,25D in the regulation of both osteoblastic and osteoclastic activity [94,95]. Both OBs and OCs express VDR [96,97], which allows 1,25D to directly affect their biological activity. Moreover, both OBs and OCs can locally synthesize the active form of 1,25D as they express CYP27B1 [96,98]. However, data obtained from in vitro studies are very heterogenic with regard to the differentiation stage of the cells (mesenchymal stem cells vs. primary OBs vs cell line), time points of treatment (2–72 h after treatment), OB origin (human/rat vs. murine), and the 1,25D concentration that was used (1–100 nM) [99,100]. This makes it difficult to compare the different studies and to draw final conclusions. Herein, we focused on the in vitro impact of VD on human OBs (hOBs), hMSCs, and human OCs (hOCs).

#### 3.2.2. Effect of 1,25D on hOBs and hMSCs

1,25D has been shown to stimulate bone formation and mineralization in all studies using hOBs, and it induced osteogenic differentiation from hMSCs. Ten nM of 1,25D stimulated the differentiation of hMSCs to OBs, and osteoblastogenesis was stimulated to a greater degree by 1,25D in hMSCs that were obtained from subjects with inadequate or deficient 25D levels than the people who were VD sufficient [87]. hMSCs, similar to hOBs and hOCs, express VDR and possess the molecular machinery for VD synthesis and metabolism, which makes them a producer and target of 1,25D [101]. Moreover, OBs express the VDBP receptors cubilin and megalin to uptake 25D [102]. In cultured primary hOBs, active VD increased the survival, differentiation, and function of these cells. Mechanisms explaining this effect include increased osteoblastogenesis [99] and inhibition of apoptosis [103,104], leading to the formation of bone nodules and bone mineralization. 1,25D has anti-apoptotic effects on primary OBs and osteoblastic cell lines by inhibiting Fas ligand-induced apoptosis and regulating components of both the Fas-related and mitochondrial apoptosis pathways [88,103]. The carefully regulated OB apoptosis plays a crucial role in healthy bone remodeling; if this process is excessive, osteocyte differentiation, bone deposition, and mineralization will all be reduced as well [105].

1,25D has been shown to increase RUNX2, small mother against decapentaplegic (SMAD) 1–3,5, osterix (OSX), ALP, and BGLAP expression in hOBs [86,88,106,107]. The other genes involved in OB proliferation and differentiation, whose expression has been shown to be increased by 1,25D, are bone morphogenetic protein-2 (BMP-2) [108] and insulin-like growth factor-binding proteins (IGFBPs): 2–4 [87,109].

The Wingless-type, Wnt-β-catenin pathway is an important regulator of OBs differentiation and function. Cytosolic β-catenin is translocated into the nucleus to stimulate osteoblastogenic gene transcription. The levels of β-catenin expression represent the functional status of the Wnt/β-catenin signaling pathway in OBs [110]. The impact of 1,25D on Wnt-β-catenin signaling in OBs was well recognized an in vitro study, where 1,25D stimulated Wnt signaling, increased β-catenin protein expression, or induced the Dickkopf-related protein 1 (DKK1) expression, leading to the intensification of calcified nodule formation in mineralized OBs [88,111,112].

Autophagy was recently recognized as an important regulator of OB survival and function. During autophagy, the toxic cytoplasmic components are removed, while nutrients are recycled to maintain cell functions and to protect against apoptosis. Impaired autophagy causes cellular dysfunction and cell death. Therefore, modulating the functional autophagy in bone cells is of therapeutic interest [113,114]. Recently, Al Saedi et al. [88] demonstrated that 1,25D may improve OB viability and function through the stimulation of functional autophagy. An additional benefit of 1,25D on the functioning of these cells could be through its effects on mitochondrial mass [88].

Vascular endothelial growth factor (VEGF) is one of the most important pro-angiogenic factors involved in the regulation of new bone blood vessel formation. Human osteoblastic cells produce VEGF, and receptors for VEGF have been identified on these cells, allowing VEGF to directly regulate survival, chemotactic migration, and OB activity [115]. 1,25D treatment increased VEGF gene expression and protein levels in primary hOBs, indicating that this hormone can exert its anabolic effects on bone by inducing angiogenesis [116,117].

In vitro experiments with primary bone cells isolated from humans demonstrate that treatment with 1,25D inhibited OB proliferation and enhanced OB maturation and mineral deposition. The expression of many genes key to OB maturation and mineral deposition are modulated by 1,25D, as has been above described [86,88]. The activation of VDR by 1,25D can exert a catabolic effect on bone mineralization to ensure serum calcium homeostasis, or it may act as a mineralization enhancer through stimulating OB maturation and the expression of genes associated with mineralization. It has been proposed that the stage of OB differentiation is one of the possible factors determining which of these two effects predominates. The phenotypically immature OBs precursors respond to 1,25D through the stimulation of RANKL expression, whereas mature OBs predominately respond through the stimulation of OC expression [118]. However, a later study by Woeckel et al. [119] demonstrated that 1,25D enhanced mineralization by the effects on hOBs in the pre-mineralization phase; it is involved in the appropriate preparation of the extracellular matrix (ECM) for mineralization. 1,25D stimulates the expression of the OB differentiation marker, ALP, and other ECM proteins, such as collagen type I (COL1A1). ALP-positive matrix vesicle production was significantly increased by 1,25D in this period of OB differentiation, and they can translocate ALP to the ECM, where ALP was incorporated to initiate mineralization [119].

In addition to the stimulation of bone formation and mineralization, 1,25D has a certain protective potential to avoid pathological over-mineralization. It may induce activin A and osteopontin (SPP1) gene expression—the recognized inhibitors of mineralization [120,121]. Moreover, a stimulator of mineralization, bone integrin-binding sialoprotein (IBSP), is inhibited by 1,25D [122].

#### 3.2.3. Effect of 1,25D on hOCs and Human Peripheral Blood Mononuclear Cells (hPBMCs)

Current evidence suggests that endogenous 1,25D synthesis and the response to this vitamin in human bone is linked with coordinated functions in both the osteoclastic and osteoblastic cells, controlling bone remodeling [123,124].

The stimulation of osteoclastogenesis by 1,25D via the OB is one of better established effects of this vitamin on OC activation. With respect to gene regulation in OBs, the 1,25D–VDR complex induces the expression of RANKL that activates RANK on OCs and their hematopoietic precursors, stimulating bone resorption through osteoclastogenesis. OPG, the soluble decoy receptor for RANKL, is repressed by 1,25D in OBs, so that the biological effect of RANKL is reinforced [124,125]. The cell-to-cell contact in combination with macrophage colony-stimulating factor (m-CSF) induces the differentiation of precursors to OCs and promotes their activity. These data indicate that OBs are the key cell responding to 1,25D with respect to OC formation [126].

In vitro studies on the effect of 1,25D on osteoclastogenesis and hOC function are conflicting, showing both stimulatory as well as inhibitory effects of this vitamin on OC differentiation and resorptive activity [89,90,96,126,127,128,129,130,131,132,133,134,135,136,137]. In 1992, Suda et al. [128] suggested that 1,25D promotes bone resorption by increasing the number and activity of osteoclasts. These effects may be direct, if the osteoclast contains the VDR and CYP27B1, and 25D promotes their differentiation in the presence of macrophage colony-stimulating factor (m-CSF) and RANKL. Kogawa et al. [129] showed that OC formation from hPBMCs in the presence of physiological concentrations of 25D resulted in significant up-regulation of the key OC transcription factor, nuclear factor of activated T cells-c1 (NFATC1), and a number of key osteoclast marker genes. An interesting observation of this study was that the OCs generated in the presence of 1,25D, although more numerous, exhibit reduced resorptive activity on hydroxyapatite-coated slides when compared to OCs that matured simply in the presence of RANKL and m-CSF [129].

The study of Zarei et al. [96] showed that the treatment of hOCs with 1,25D significantly suppressed the expression of osteoclast fusion markers NFATC1 and transmembrane 7 superfamily member 4 (TM7SF4), reduced OC size, but increased OC number and resorptive activity. An increase in osteoclast resorption was due to less fusion, resulting in more small OCs in 1,25D-treated samples, as a few larger multinucleated OCs were observed in the control samples. Sakai et al. [130] also demonstrated that 1,25D treatment significantly inhibited the expression of NFATC1 in hOCs by upregulating the expression of interferon-beta, which is a strong inhibitor of osteoclastogenesis. However, the suppression of NFATC1 resulted in significantly inhibited hOC formation, which is opposite to the finding of Zarei et al. [96]. A similar effect of 1,25D treatment on mature multinucleated osteoclasts obtained from human monocytes was observed by Allard et al. [89], who demonstrated that 1,25D significantly inhibited osteoclastogenesis at early stages but had no effect on osteoclast-mediated bone resorption activity. Kudo et al. [131] also noticed that 1,25D did not stimulate resorptive activity of hOCs formed from cultures of hPBMCs. They suggested that it was more likely that 1,25D could influence OC activity indirectly.

Kim et al. [132] examined the direct effects of 1,25D on the osteoclastogenesis of human peripheral blood osteoclast precursors. They showed that 1,25D suppressed the expression of RANK in the hOC precursor and strongly inhibited OC differentiation. The mechanism responsible for the inhibition of RANK by this vitamin was a down-regulation of the c-Fms, the receptor of m-CSF, which is required for RANK expression. In line with the above observation, the treatment of PBMCs from healthy donors with 1,25D dose-dependently suppressed osteoclastogenesis in vitro, as has been shown by the reduced number of TRAP-positive OCs [133].

Wnt ligand 10b (Wnt-10b) is a key pathway for bone formation through increases in the number of OBs and the rate of mineral apposition [134]. A recent study by Lu et al. [135] demonstrated that in primary cell cultures of OCs, calcitriol increased Wnt-10b expression, but in parallel, it reduced the OCs fusion ability, the number of TRAP-positive OCs, as well as their bone-resorbing activity. This finding is compatible with higher Wnt-10b levels and lower TRAP-5b activity in HD patients receiving calcitriol compared with patients not taking this vitamin [135]. Although both hOBs and hOCs may be the source of Wnt-10b, the therapeutic dose of calcitriol enhanced Wnt-10b secretion only from OCs in this study. Taken together, the bone anabolic effect of a therapeutic dose calcitriol can promote OB function and it can inhibit OC maturation and resorbing capacity both in OC cultures in vitro and in hemodialyzed patients in vivo [135].

Autophagy has been reported to increase the number and function of OCs [135,137]. The recent study by Ji et al. [90] proved that 1,25D may be a strong regulator of autophagy in OCs, and it had a dual effect on osteoclastogenesis this way. 1,25D can directly (without RANKL) suppress OC precursor autophagy, which negatively regulates the proliferation of these cells. However, 1,25D can indirectly upregulate the autophagy response of OC precursors, thereby enhancing OC formation in the presence of RANKL.

**Figure 4:**
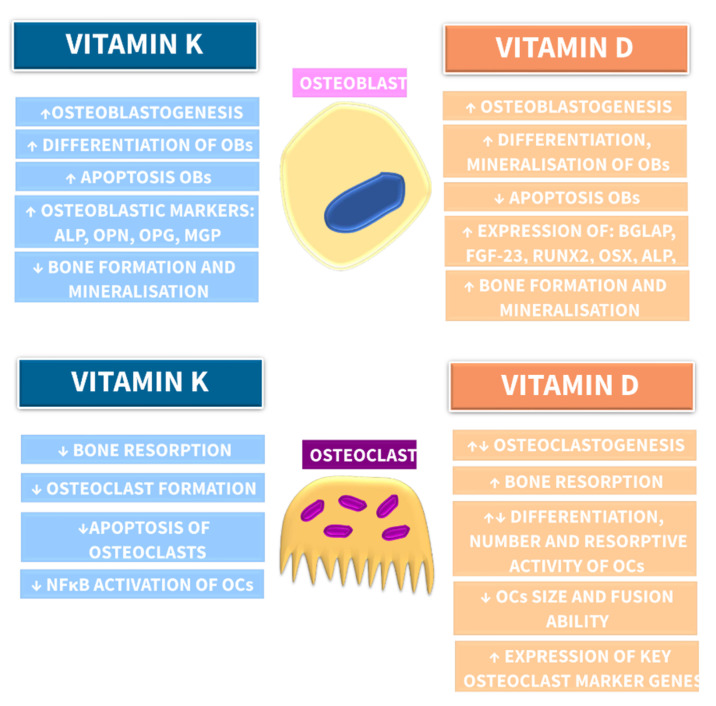
Impact of vitamin K and vitamin D on bone remodeling—evidence derived from in vitro studies. Abbreviations: OBs, osteoblasts; ALP, alkaline phosphatase; OPN, osteopontin; OPG, osteoprotegerin; MGP, matrix Gla proteins; BGLAP, bone-Gla-protein; FGF-23, fibroblast growth factor 23; RUNX2, Runt-related transcription factor 2; OSX, osterix; NF-*κ*B, nuclear factor kappa-light-chain-enhancer of activated B cells; OCs, osteoclasts; ↑, increase; ↓, decrease.

Taken together, the in vitro studies revealed that 1,25D may function to optimize osteoclastogenesis, but on the other hand, it can mitigate hyperactive osteoclastic resorptive activity. The main effects of VK and VD on bone cells derived from in vitro studies are summarized in Figure 4.

## 4. Vitamin K and Vitamin D in Bone Remodeling—In Vivo Studies

### 4.1. Vitamin K and Bone Remodeling—In Vivo Animal Models

According to our best knowledge, there is only one report examining the influence of VK2 on cortical bone mass and bone strength in rats with renal insufficiency. This study demonstrated that the administration of VK2 increased cortical bone strength without changing bone mineral density (BMD) in nephrectomized rats [138], suggesting that VK could affect bone integrity without altering BMD.

However, several animal models of osteoporosis have been used to study the effects of VK on bone metabolism. Table 2 reports the main in vivo studies with VK and its association with bone remodeling. The treatment of ovariectomized [139,140], unilaterally sciatic neurectomized [38], and tail suspended rats [141] with VK found positive effects on bone health. Histologic and microcomputed tomographic evaluations demonstrated that VK2 supplementation inhibited the loss of bone mass as well as improved OB function and bone architecture. Kim et al. [142] observed that VK administration in high-fat diet mice resulted in an increase in bone formation and a reduction in bone resorption. Some animal studies investigated the effect of the coadministration of VK2 and other bone acting drugs on osteoporosis. The coadministration of VK2 and Teriparatide improved OB function and increased Gla-OC serum levels [140]. The effect of the combined use of VK2 and bisphosphonate showed that VK2 could ameliorate the suppressive effect of bisphosphonates on bone turnover and increase bone volume as well as the bone formation parameters [143]. Combining VK2 with VD_3_ showed an additional protective effect on osteoporosis versus VK2 treatment alone [144]. In addition, the combined effect of VK and antiresorptive drugs on bone mechanical strength were assessed, providing contrasting results. Otomo et al. [145] did not observe any effects of K2 supplementation with Risendronate on bone quality, while Matsumoto et al. [146] showed that MK-4 treatment enhanced the positive effect of Risendronate on bone strength. These observations suggest that the combined administration of VK with other osteoprotective drugs may exert a more promising effect on bone health than VK alone.

In general, evidence from in vitro and animal studies supported the role of VK2 in bone health, suggesting a potential benefit for its use in patients with osteoporosis.

### 4.2. Vitamin D and Bone Remodeling—In Vivo CKD Animal Models

Despite widespread clinical use, there are limited studies on animal models of CKD that examine the bone tissue material and structural properties after VD (or its analogs) treatment, and the obtained results are inconclusive. Table 2 shows the main in vivo studies with VD and bone remodeling. One of the first studies, performed on female dogs with a 5/6 nephrectomy showed that the oral administration of 20 μg of 25D, three times a week, prevented secondary hyperparathyroidism and morphologic abnormalities associated with renal osteodystrophy during a two-year observation period [150]. Jablonski et al. [151] also demonstrated that rats that underwent a 5/6 nephrectomy, treated three times a week for 3 months with 0.17 μg/100 g body weight (BW) of calcitriol, exhibited higher trabecular volume, lower eroded surface and osteoid surface compared to untreated animals. A cross-sectional analysis showed that with 1,25D treatment, the inner femoral shaft diameter, femoral widths, bone stiffness, and time to fracture were normalized [151].

In the study by Jokihaara et al. [147], 5/6 nephrectomized rats obtained paricalcitol at a dose of 100 ng/rat, 3 times per week for 12 weeks. The femoral neck BMD and mechanical properties were higher than in untreated CKD animals, while no beneficial effects were observed in BMD or mechanical properties at the femoral diaphysis. The treatment of female rats subjected to a 7/8 nephrectomy with calcitriol at a dose of 10 ng/kg BW, 5 times per week for 8 weeks, proved to have a positive effect on bone microarchitecture, achieving normal trabecular connectivity [148]. Lu et al. [135] explored the effects of calcitriol on bone microarchitectures in CKD mice, using the 5/6 nephrectomy model, which were treated orally with 25 or 150 IU/kg/day of calcitriol. The bone volume fraction increased in mice treated one month with a higher dose of 1,25D; however, trabecular thickness was not significantly different in any group. The average cortical thickness was higher, whereas cortical porosity was lower in CKD animals treated with 150 IU/kg/day of calcitriol than in untreated CKD mice. Although there was no change in femoral BMD between the studied groups, the authors concluded that calcitriol, especially in the higher dose, can promote the growth of both trabecular and cortical bone in CKD.

The effect of daily or intermittent calcitriol administration in enhancing bone growth in CKD was studied by Sanchez et al. [149]. The animals were treated daily or thrice weekly with calcitriol for 4 weeks, but the total weekly dose of calcitriol was the same (350 ng/kg/week). Although calcitriol increased the serum calcium, it did not lower PTH or improve tibia and body length. However, calcitriol was effective in enhancing chondrocyte maturation and restoration of the growth plate architecture. Moreover, RANKL levels were improved with calcitriol treatment without changes in OPG, suggesting an enhancement of chondroclastogenesis and mineralization.

Newman et al. [152] used a rat model of progressive CKD (Cy/+), which is characterized by autosomal dominant cystic disease. Starting at 25 weeks of age, Cy/+ male rats were treated with 10 ng/kg BW of calcitriol, intraperitoneally, 3× weekly for 5 weeks. Apart from a significant suppression of PTH levels in animals with CKD, 1,25D had no impact on cortical or cancellous bone volume, bone turnover, OC number, or whole bone mechanical properties.

In contrast to the above data, some studies reported that 1,25D therapy can lead to bone turnover alteration and a reduction of cortical thickness in CKD rats. Male subtotally nephrectomized Sprague–Dawley rats were treated with 0.25 μg/kg/day of calcitriol starting 2 weeks after subtotal nephrectomy and continued for the next 14 weeks. In rats treated with 1,25D, a dramatically increased bone formation rate, an irregular osteoid deposition, and chaotic mineralization were observed. The dynamic bone histomorphometric parameters could not be measured in these animals due to the chaotic tetracycline incorporation. An excessive amount of osteoid, in combination with reduced bone resorption, led to a high bone area, which was improperly mineralized in rats treated with 1,25D. Moreover, kidney function was significantly more impaired, whereas aortic calcification was increased in rats treated with calcitriol compared to the CKD group [153].

Bisson et al. [154] treated rats with a 5/6 nephrectomy with 0.5 μg/kg BW of 1,25D, 3 times per week for 6 weeks using a high calcium and phosphate diet. Healthy rats on a standard diet, healthy rats with 1,25D on a high calcium and phosphate diet, as well as 5/6 nephrectomized rats on a standard diet were also included in this study. Cortical bone volume and area were significantly reduced in both CKD groups as compared to healthy controls; however, cortical bone thickness, the inner and outer cortical perimeter, and cortical bone mineral content were the most reduced in CKD rats treated with 1,25D on a high calcium and phosphate diet as compared to the other groups. The trabecular bone volume, trabecular thickness, trabecular number, osteoid volume, and osteoid thickness were significantly increased in these animals. Dynamic bone parameter analysis revealed a lower mineralization surface, bone formation rate, mineral apposition rate, and OC number in CKD treated with 1,25D on a high calcium and phosphate diet compared to the other groups. This study indicated that despite low PTH levels, treatment with calcitriol combined with a high calcium and phosphate diet induced low bone turnover and mineralization defects, which is likely explained by the high calcitriol dose [154].

Summarizing, the recently performed studies showed that the treatment of CKD animals with 1,25D may not improve bone quality [152], or even can be associated with mineralization defects [153,154]. These observations are consistent with the results of studies performed on non-CKD animals [155,156,157], which reported that treatment with calcitriol led to growth plate defects, an accumulation of osteoid and prolongation of mineralization lag time, reduction of cortical thickness, and suppression of bone matrix mineralization. Interestingly, VDR−/−, 1α-hydroxylase −/−, or double mutant mice on a rescue diet showed reduced bone formation, which is corrected by 1,25D, indicating a physiological anabolic role for the endogenous VD mediated by VDR in vivo [158,159]. Thus, it is possible that pharmacological, but not physiological, doses of 1,25D markedly increase RANKL expression by OBs, stimulating cortical osteoclastogenesis and bone resorption [94].

Such divergent data obtained during the treatment of renal osteodystrophy with 1,25D may result from the use of different animal species, doses, and treatment regimens, a differential degree of PTH reduction, and possibly distinct effects of this vitamin in different skeletal sites. Nevertheless, data obtained from animal models indicated that 1,25D may have a direct effect on bone, independent of its effect on PTH suppression.

## 5. Vitamin K, Vitamin D, and Bone Health in Patients with CKD—Clinical Evidence

### 5.1. The Impact of Vitamin K on Bone Health in Patients with CKD

Studies regarding the association between poor VK status and bone metabolism, BMD, and the risk of fracture in CKD patients are limited. The main results obtained using VK supplementation and its association with bone health are summarized in Table 3. A small but growing number of recent studies have consistently suggested that there is an association between poor VK status in CKD patients and bone health [40,160,161,162,163].

Kohlmeier et al. [160] were the first to demonstrate an independent association between poor VK1 status and risk of bone fracture in HD patients. In the VIKI study, total OC and ucOC levels were higher in patients with CKD than in healthy controls, and over 50% of HD patents had vertebral fractures. Additionally, in this observational study, VK1 deficiency was the strongest independent predictor for vertebral fractures in these patients [56]. In another study by Fusaro et al. [161], HD patients treated with warfarin (an antagonist of VK) had an increased risk of vertebral fractures compared to those without warfarin treatment. These studies suggest that the VK axis is important in preserving bone mass. Evenepoel et al. [162] observed an independent association between VK status and bone health. Data from this study showed that the high dp-ucMGP levels were independently correlated with low BMD and incident of fractures, whereas no associations were observed between VK status and bone turnover markers in patients with end-stage renal disease (ESRD). Additionally, poor VK status at the time of renal transplantation can be considered as a risk factor for incident fractures.

Studies on the effect of clinical VK supplementation on BMD are also scarce. Sasaki at al. [163] showed that MK-4 supplementation for a year in steroid-treated patients with glomerulonephritis prevented steroid-induced bone loss. The patients on hemodialysis supplemented with MK-7 showed a decrease in dp-ucMGP, ucOC, and PIVKA-II, implicating that MK-7 improves VK status in the liver, bone, and vasculature [30,169,170].

To date, very little is known about the VK insufficiency and bone remodeling in CKD. Holden et al. [3] performed a study on patients with stage 3–5 CKD and found that high serum ucOC levels were positively associated with phosphate and PTH, whereas it was inversely associated with 25D levels, suggesting a relationship with bone remodeling [9]. Moreover, 6%, 60%, and more than 90% of patients in this study met the criteria for subclinical VK insufficiency, regarding VK1, ucOC, and PIVKA-II levels, respectively. Voong et al. [37] showed that subclinical VK deficiency is common in patients on dialysis, but it is also more frequent with worsening renal function in those CKD patients not yet on dialysis.

So far, VK supplementation and bone fractures in CKD patients have not been studied in a controlled, randomized clinical trial. Currently, there is one randomized controlled double-blind trial, RenaKvit, being performed in Denmark to address the effect on VK2 (MK7) on cardiovascular and bone disease in CKD patients. This study is evaluating the impact of VK2 supplementation on arterial stiffness and bone mineral density in HD patients. The RenaKvit trial is evaluating the impact of 360 μg of VK2 during a period of 2 × 12 months [171]. Increasing evidence that VK is also involved in vascular health is supported by controlled, randomized trials [14,172,173]. Oikonomaki et al. [174] investigated 1-year supplementation of 200 μg of VK (VK2/MK-7) in the prevention of VC progression among HD patients and found reduced serum uc-MPG levels, but they did not observe significant effects on the regression of VC. There are still ongoing trials evaluating the influence of oral VK supplementation on VC in HD patients—with 5 mg VK1 [172] and 360 mcg VK2, 3 times weekly for 18 months. Other trials that are in process include Vitamin K supplementation in patients on hemodialysis (VISTA) in phase 2, with 400 mcg of VK1 three times a week, on dialysis days for four months [175]; Evaluation of Vitamin K Supplementation for Calcific Uremic Arteriolopathy (VitK-CUA) with administration of 10 mg of VK1 three times a week after dialysis for 12 weeks [176]; Comparative Study Evaluating the Effect of Vitamin K1 Versus Vitamin K2 on Vascular Calcification in Dialysis Patients in phase 2, with 10 mg of VK1 thrice a week for 3 months and phase 3 with 90 μg per day of VK2 [177].

### 5.2. Impact of Vitamin D on Bone Health in Patients with CKD

Together with the declining kidney function, many abnormalities concerning 1,25D, FGF-23, and PTH levels were observed. A disruption of the delicate balance between 1,25D, calcium, phosphorus, and PTH lead to secondary hyperparathyroidism and increased risk of bone disease. Several studies showed an inverse association between VD deficiency/insufficiency and PTH levels [178,179,180,181,182]. Metzger et al. [182] observed that serum PTH levels rise steeply when 25D values fall below 8 ng/mL; on the contrary, a mild decrease in this hormone concentration was seen when 25D levels exceeded 20 ng/mL. These observations are in line with other studies [183,184,185], suggesting that PTH increases significantly when 25D levels in CKD patients are below 30 ng/mL. Some authors noticed that VD deficiency was associated with lower values of serum calcium [179,186], which may be an additional cause of secondary hyperparathyroidism.

Studies showing an association between VD and BMD or bone fractures in the CKD population are limited. A summary of the key findings is presented in Table 3. A retrospective study conducted by Coen et al. [164] demonstrated that patients with low 25D levels (<15 ng/mL) had a lower bone formation rate and trabecular mineralization surface. Another retrospective study [165] showed that HD patients with fractures had a significantly lower VD concentration in comparison to patients without fractures, and low VD levels were associated with reduced BMD. Additionally, low levels of VD were independently related to increased fracture risk. Other studies showed that patients with lower 25D levels had increased subperiosteal resorption, reduced BMD, and increased skeletal fractures [187,188,189]. Interestingly, ESRD patients showed radiologic features of secondary hyperparathyroidism [188]. In line with these results is the Korean National Health and Nutrition Examination Survey [190], which reported that the BMD of CKD patients was lower in those with serum 25D < 50 nmol/L than in patients within serum 25D > 50 nmol/L. On the contrary, Brunerová et al. [191] did not demonstrate significant differences in trabecular bone and T-scores in HD patients with regard to their 25D levels. Based on these studies, it seems that low VD status is associated with an increased risk of fractures due to mineralization defects and lower BMD.

The optimal management of CKD-MBD is a daily challenge for nephrologists. VD supplementation is required for CKD patients to suppress PTH increases as well as to correct abnormalities of bone and mineral metabolism. In daily practice, VD (cholecalciferol or ergocalciferol) can be used in daily, weekly, or monthly doses. On the other hand, when VD supplementation is ineffective, therapy with VDRA (calcitriol, paricalcitol, doxercalciferol, alfacalcidiol) can be initiated [65]. Nevertheless, the issue of which form of VD should be used in patients with CKD is still a matter for debate. Current guidelines propose that CKD patients with VD deficiency should receive supplementation using the same recommendation as the general population [2,77,192]. The KDOQI recommend a dosage of 1000–2000 IU of VD_3_ for VD repletion, but it confirmed that some patients with CKD may require a more aggressive therapeutic strategy [77]. However, irrespective of the chosen form of VD, it is worth emphasizing that when serum 25D levels are greater than 100 ng/mL, the risk of hypervitaminosis D toxicity can occur, including adverse effects such as hypercalcemia and hyperphosphatemia [65]. According to KDOQI 2017 recommendations, “mild and asymptomatic hypocalcemia can be tolerated in order to avoid inappropriate calcium loading in adults”. The KDOQI work group holds the view that avoidance of hypercalcemia will protect vascular and valvular calcifications, arrhythmia, and an increased risk for cardiovascular events in adults with CKD. In contrast to adults, the KDOQI work group endorses the recommendation to maintain serum calcium concentrations in children with CKD in the age-appropriate normal range, because the growing skeleton must be in positive calcium balance to achieve normal bone accrual. In this age group, the permissive mild hypocalcemia may have deleterious effects on skeletal integrity and should be avoided [193]. The excessive VC can also be caused by hyperphosphatemia (especially in the setting of persistent hypercalcemia) and a positive net phosphate balance. As has been recently summarized by Cozzolino et al. [194], hyperphosphatemia can cause damage in several cells and tissues, among others in the heart and blood vessels, where it is strongly associated with vascular and valvular calcification, arteriosclerosis, and an increased risk of cardiovascular death, especially in advanced CKD patients.

Vitamin D analogs (VDRAs), which are less calcemic and phosphatemic than the active form of vitamin D, are becoming the standard for the treatment of secondary hyperparathyroidism. The experimental models and clinical studies suggest that VDRAs can promote VC probably only at high doses if they induce or exacerbate hyperphosphatemia, while the use of these agents in more physiological doses (just enough to correct secondary hyperparathyroidism) might even be protective against VC [195,196,197].

In a study by Oksa et al. [198], a 12-month cholecalciferol supplementation of 5000 or 20,000 IU/week significantly improved VD deficiency, increased calcidiol, and less markedly, calcitriol levels, and decreased PTH levels without adverse effects on serum mineral parameters. Additionally, the number of hypercalciuric patients increased with a higher VD dose, although there was no sustained rise in calcuria. A similar decrease in PTH levels, following cholecalciferol supplementation, had also been presented in other studies [199,200,201]. Additionally, Yadav et al. [201] reported that cholecalciferol supplementation not only suppressed secondary hyperparathyroidism but also favorably changed the biochemical parameters of mineral metabolism in patients with CKD. On the contrary, supplementation with 50,000 IU cholecalciferol weekly [202] or 1000 IU cholecalciferol daily [203] found no difference in PTH levels in CKD patients. Post-hoc analysis of the Vitamin D, Calcium, Lyon Study II (DECALYOS II) study [166] reported that daily supplementation of 800 IU of cholecalciferol in combination with 1200 mg of calcium significantly increased serum 25D concentrations and radius BMD in an elderly woman with moderate CKD and severe VD deficiency. On the other hand, Mager et al. [167] found no significant differences in FGF-23, OC, N-terminal telopeptide (NTx), and BMD as measured by dual X-ray absorptiometry (DXA) after daily (2000 IU/day) and monthly (40,000 IU/month) VD_3_ supplementation for six months in adults with diabetes mellitus and CKD. Summarizing, all the above-mentioned randomized studies demonstrated that a correction of VD deficiency with cholecalciferol supplementation led to the efficient achievement of a sufficient level of 25D in CKD patients.

In another study, Moe et al. [168] compared cholecalciferol at a dose of 4000 IU/d for a month, and then 2000 IU/d for two months and doxercalciferol at a dose of 1 µg/d for 12 weeks. The PTH levels decreased by 10% and 30% in the cholecalciferol and the doxercalciferol groups, respectively. However, there was no significant difference in the mean change between these two treatments. Additionally, there were no increases in serum calcium and urinary calcium/creatinine in the cholecalciferol group, whereas in the doxercalciferol-treated patients, there was a slight increase in the serum calcium level and urinary calcium exertion level. In the next study by Zelnick et al. [204], patients received either cholecalciferol (4000 IU daily for 1 month and then 2000 IU daily for 5 months) or calcitriol (0.25 µg daily for 1 month and then 0.5 µg daily for 5 month). There was no difference in PTH levels in both groups, and only the calcitriol-treated group showed a significant change in FGF-23 levels. Interestingly, both groups significantly increased circulating 24,25D concentrations and the ratio of 24,25D/25D.

Studies regarding VD supplementation, using ergocalciferol in CKD patients with VD insufficiency, have shown effective correction of 25D [205,206,207,208,209,210]. Treatment with ergocalciferol among patients with stage 3 CKD resulted in a significant decrease of serum PTH concentrations [207,208]. Similarly, Zisman et al. [205] observed a decrease in PTH levels but only in CKD stage 3. On the contrary, Porter et al. [206] and Gravesen et al. [209] did not find any differences in PTH levels and bone/mineral parameters. Wetmore et al. [211] compared the efficacy of cholecalciferol and ergocalciferol in the CKD population, and they showed that therapy with cholecalciferol is more effective at raising serum 25D concentration, suggesting that cholecalciferol may be more effective. These results are in line with other studies conducted on CKD patients [212,213].

## 6. Impact of Combined Vitamin K and Vitamin D Supplementation on Bone Health in Patients with CKD

**Figure 5:**
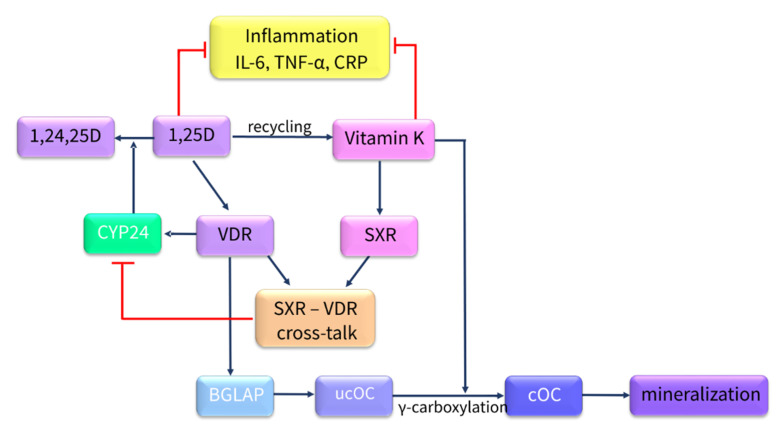
Potential synergy between vitamin K and vitamin D action in bone. Abbreviations: IL-6, interleukin 6; TNF-α, tumor necrosis factor α; CRP, C-reactive protein; 1,24,25D, 1,24,25-trihydroxyvitamin D; 1,25-dihydroxyvitamin D; CYP-24, 25-hydroxyvitamin D-24α-hydroxylase; VDR, vitamin D receptor; SXR, steroid and xenobiotic receptor; BGLAP, bone-Gla-protein; ucOC, uncarboxylated osteocalcin; cOC, carboxylated osteocalcin.

A growing body of evidence from in vitro [83] and in vivo studies [144], as well as clinical trials [214,215,216,217], showed that bone metabolism depends on the interaction between vitamins D and K, as has been schematically presented in Figure 5. However, the interplay between these vitamins in relation to bone health remains not fully elucidated, especially in CKD.

OC is produced by OBs during bone formation. It is one of the most abundant proteins in bone and is necessary for bone mineralization. The synthesis of fully functional OC and its expression are controlled by both VK and VD [218]. 1,25D is a known promoter for OC gene expression [219], whereas VK is acquired for proper γ-carboxylation of OC, thereby increasing its beneficial effect on bone formation [220,221]. Fully carboxylated OC is positioned into hydroxyapatite and strongly binds calcium, providing bone mineralization [222]. Moreover, 1,25D is able to regulate the γ-carboxylation of OC, decreasing ucOC secretion in human osteosarcoma cells [223].

VD can exert an anabolic effect in bone through increasing OB activity and reducing OC activity [224]. Koshihara et al. [225] demonstrated that VK2 promoted α25D-induced mineralization in human periosteal OBs. Similarly, the study by Poon et al. [83] showed that the coadministration of VK and VD caused an enhancement of calcium deposits and additionally increased the levels of bone anabolic markers of bone formation in the OBs of obese/diabetic mice. These findings suggest the synergistic effect of both vitamins in relation to bone formation and mineralization.

On the other hand, there are suggestions that excessive amounts of VD can increase VK requirements, inducing a relative VK deficiency by direct stimulation of the synthesis of VK-dependent proteins [226,227].

Another field of VD and VK cooperation is inflammation, which is casually implicated in osteoporosis [228]. VK is related to a decreased production of inflammatory markers: C-reactive protein, isoprostanes, and proinflammatory IL-6 [229,230,231,232]. VD exerts several immunomodulatory functions, such as the suppression of pro-inflammatory cytokine expression and regulation of immune cell activity [233]. VD supplementation reduced tumor necrosis factor α (TNF-α) levels in patients with osteoporosis [234].

VK and VD also overlap metabolically at the cellular level. The VK cycle is a source of electron transfer for antioxidant power in hOBs, and 1,25D can enhance the reductive recycling of MK4 [220]. SXR, which can be activated by VK2, was shown to be able to crosstalk with VDR, and this way deranging 1,25D metabolism. It was shown that the SXR-VDR crosstalk can inhibit VDR-mediated CYP24 promoter activity [235]. CYP24-mediated hydroxylation of 1,25D is a critical step in its catabolism, and it appears to be responsible for controlling systemic 1,25D levels. CYP24 is directly regulated by VDR, and it is expressed mainly in the kidney, where VDR is also abundant. However, recent studies [5,6,7] have shown that bone cells also have molecular machinery capable of producing and metabolizing 1,25D, suggesting that such an interaction may be present in bone as well. Therefore, VK and VD can mutually intensify each other’s metabolism.

On the other hand, the activation of SXR in the liver can lead to the down-regulation of CYP2D25, which is an enzyme involved in 1,25D biosynthesis [236] that may be related to osteomalacia.

In vivo evidence also reported that combined VD and VK2 supplementation prevented bone loss by stimulating OC production in ovariectomized (OVX) rats [144,224,237,238], whereas no effect was observed when these vitamins were given separately [238]. A recently published study [239] demonstrated the beneficial effects of eggshell calcium, VD_3_, and VK2 on the inhibition of OVX-induced bone loss in rats. The combination of these three elements increased cortical and trabecular bone quality as well as improved biochemical and densitometrical parameters. Meanwhile, Iwamoto et al. [240] found no synergistic effect of VK and VD on intestinal calcium absorption, renal calcium reabsorption, and cancellous and cortical bone mass in calcium-deficient rats. Therefore, it seems that the availability of calcium is an important factor in determining the synergistic effect of these vitamins in relation to bone mineralization.

The above presented data suggest that combined VD and VK supplementation may be beneficial for bone health in the course of CKD. Unfortunately, there have been no randomized, controlled trials that examined the effects of such a combination in this population. Many studies have investigated the combined effect of VK and VD supplementation on skeletal integrity in the general population, especially in postmenopausal women with osteoporosis [214,215,216,217,241]. Nevertheless, these studies did not provide consistent conclusions, and the effect of the coadministration of these vitamins is still poorly understood. A recently published meta-analysis [241] was based on eight selected randomized controlled trials, which evaluated the combined effect of supplementation with VK and VD on bone quality. This meta-analysis showed that the simultaneous administration of VD and VK can improve bone quality by increasing the total and third lumbar BMD and decreasing ucOC. However, in the remaining lumbar segments and femoral neck, the combined supplementation of these vitamins did not significantly increase BMD. Taken together, this report indicates that the coadministration of VD and VK can have a more favorable effect on bone health than giving each one separately. However, the authors emphasize that conclusions from this meta-analysis should be interpreted with caution due to potential publication bias.

Taken together, despite the growing body of evidence from in vitro and in vivo studies, as well as clinical trials, the synergy between VK and VD in relation to bone quality and quantity remains not fully elucidated. Therefore, further studies are needed to explain the exact mechanisms of the combined effects of these vitamins on bone health, especially in the CKD population.

## 7. Conclusions

The current understanding is that patients with CKD are a clinical group at high risk for VD and VK deficiency. Therefore, these patients are prone to suffering from many consequences of VK and VD deficiency, such as poor bone health and a higher risk of fractures. Therefore, finding new solutions for the prevention/treatment of osteoporosis in this population is a particular challenge. The majority of randomized-controlled studies performed on osteoporotic patients without CKD suggest that combined VK and VD supplementation may be more beneficial for the prevention and treatment of bone loss. Moreover, supplementation with these vitamins is easily accessible, safe to use, and relatively inexpensive. However, there is still not enough evidence to recommend VK and VD supplementation. Based on studies in the general population, we can suspect that VD and VK supplementation in CKD patients may be a possible therapeutic target for improving bone health. Due to the lack of adequate clinical studies in this population, the question arises whether CKD patients might benefit from simultaneous VK and VD supplementation. This creates a need for further research, in which an investigation of the potential synergistic effect of combined supplementation of VD and VK on bone health in this population should receive more attention.

## Figures and Tables

**Table 1:** Role of vitamin K and vitamin D in bone remodeling in chronic kidney disease (CKD): pre-clinical evidence.

Reference	Model	Dose	Results
**Vitamin K**			
[79]	MC3T3‑E1 osteoblasts cell line	VK2 (10^−8^–10^−3^ M) for 1–5 days VK2 (10^−5^, 10^−6^ and 10^−7^ M) for 24 h on days 1, 3, 5 and 7	VK2 promoted osteoblast differentiation and mineralization, induced autophagy in osteoblasts
[28]	The human cell lines HOS, MG-63, Saos-2, LS180, and HeLa	VK2	VK2 activates SXR and induces expression of the SXR target gene; VK2 treatment of osteosarcoma cells increased mRNA levels of OB: ALP, OPG, OPN, and MGP
[85]	Bone marrow cells were isolated from male Wistar rats (3 weeks old)	MK-7 (10^−8^–10^−5^ M)	MK-7 can inhibit osteoclastic bone resorption; MK-7 has an inhibitory effect on the bone-resorbing factors-induced decrease in bone calcium content
**Vitamin D**			
[78]	Bone marrow cells from the femur from elderly patients with type II osteoporosis	10 nM 1,25D and 0.5, 1.0, 2.5, 10 µM MK-4 or VK1	MK-4 and VK1 inhibited 1,25D-induced osteoclast formation and promoted the differentiation of bone marrow cells; MK-4 and VK1 decreased the RANKL and enhanced OPG
[86]	iliac crest bone biopsy samples from 11 paediatric dialysis patients	8 months of doxercalciferol therapy (an average of 19.3 ± 3.8 mcg of doxercalciferol per week)	1,25D increases the maturation of OBs lineage cells, stimulates osteocyte apoptosis and increases RANKL/OPG expression, increases the number of osteocytes
[87]	hMSCs from 53 subjects scheduled for hip arthroplasty	10 nM 1,25D	1,25D stimulated the differentiation of hMSCs to OBs; greater stimulation of in vitro osteoblast differentiation by 1,25D in hMSCs from younger subjects, and who had serum 25D ≤ 20 ng/mL
[88]	Primary OB cells, with a pre-osteoblastic phenotype from healthy male donors	1,25D (10^−8^ M)	1,25D increased differentiation, mineralization and survival of osteoblasts
[89]	Monocytes from blood of healthy adult volunteer donors	VD (25D-100 nM and 1,25D-5 nM)	1,25D inhibits osteoclastogenesis
[90]	The tibia from 4-week-old littermate C57BL/6J mice		1,25D can directly (in absence of RANKL) suppress OC precursor autophagy, which negatively regulates the proliferation of these cells; 1,25D can indirectly upregulate the autophagy response of OC precursors, thereby enhancing OC formation in the presence of RANKL
**Vitamin D and Vitamin K**			
[83]	Primary osteoblasts harvested from the iliac crests of C57BL/KsJ lean (+/+) and obese/diabetic (db/db) mice	VK2 (10 nM) and 1,25D (10 nM) alone and in combination	The combined use of VK2 and 1,25D enhanced calcium deposits formation in OBs and increased the levels of bone anabolic markers and bone formation transcription factors

Abbreviations: VK2, vitamin K2; HOS, human osteosarcoma cell line; SXR, steroid and xenobiotic receptor; OB, osteoblast; ALP, alkaline phosphatase; OPG, osteoprotegerin; OPN, osteopontin; MGP, matrix Gla protein; MK-7, menaquinone-7; VK1, vitamin K1; 1,25D, 25-dihydroxyvitamin D; RANKL, Receptor Activator for Nuclear Factor κ B Ligand; hMSCs, human bone marrow stromal cells; 25D, 25-hydroxyvitamin D; VD, vitamin D; OC, osteocalcin.

**Table 2:** Vitamin K and vitamin D in bone remodeling—in vivo studies.

Reference	Model	Dose	Results
**Vitamin K**			
[138]	*n* = 30, male Sprague–Dawley rats; assigned to three groups: sham operation (control), 5/6 nephrectomy and 5/6 nephrectomy + oral VK2	VK2 (menaquinone-4, menatetrenone): 30 mg/kg, 5 days/week	The administration of VK2 increased cortical bone strength without changing bone mineral density (BMD) and improved renal function.
[140]	*n* = 25, OVX female Sprague–Dawley rats assigned to five groups: the sham, ovariectomy (OVX), VK, TPTD and VK + TPTD	VK (menaquinone-4): 30 mg/kg/day TPTD: 30 µg/kg, 3 times/week	The coadministration of VK2 and TPTD improved OB function and the OB surface, and increased Gla-OC serum levels, improved the BMD and bone strength of the femur.
[142]	*n* = 42, male C57BL/6J mice divided into six groups: normal diet, normal diet + VK1, normal diet + VK2, 45% high-fat diet, 45% high-fat diet + VK1, a 45% high-fat diet + VK2	VK1 and VK2: 200 mg/1000 g	VK administration in high-fat diet mice resulted in an increase in bone formation and a reduction in bone resorption.
[143]	*n* = 30, male Sprague–Dawley rats assigned to five groups: nonsuspended group, tail-suspended group with vehicle alone, tail-suspended group with VK2, tail suspended group with bisphosphonate, tail-suspended group with combination of bisphosphonate and VK2	Bisphosphonate (incadronate): 0.1 mgP/kg body weight VK2: 24 mg/kg body weight/day	The effect of combined use of VK2 and bisphosphonate showed increased bone volume without supressing bone turnover.
[146]	*n* = 59, female ICR mice after sham-operated or ovariectomized; OVX divided into six groups: treated with risedronate (R), MK-4 (K), R+K, either the treatment was withdrawn or switched to K or R in the case of R and K	Risedronate: 0.25 mg/kg/day VK2: 100 μg MK-4/kg/day	Prior 8-week treatment with MK-4 followed by the 8-week risedronate significantly increased femur strength.
**Vitamin D**			
[147]	*n* = 45, rats assigned to sham-operation or 5/6 nephrectomy surgery (NTX): divided into two groups: the untreated NTX and NTX + paricalcitol.	1500 IU/kg VD; for the 12-week: paricalcitol:100 ng/rat, 3 times per week	Paricalcitol efficiently ameliorates advanced renal insufficiency induced loss of mineral and mechanical competence of rat bones, prevented the renal impairment associated decrease in vBMD at the femoral neck and cBMD at the femoral midshaft, and restored bone strength at the femoral neck
[148]	*n* = 49, female Sprague–Dawley rats after 7/8 nephrectomy and CKD + OVX group; CKD + OVX were divided into 6 groups: placebo, E2 (10 μg/kg/day), E2 (30 μg/kg/day), calcitriol (10 ng/kg/day), E2 (10 μg/kg/day) + calcitriol, E2 (30 μg/kg/day) + calcitriol	Calcitriol:10 ng/kg BW, 5 times per week for 8 weeks	Calcitriol reduces bone loss but also improves trabecular connectivity; combined treatment with E2-30 + calcitriol was capable of achieving normal trabecular bone volume, trabecular remodeling, and connectivity
[149]	*n* = 36, 5/6 nephrectomized male Wistar rats divided into groups: control, Nx-Int D, Nx-Daily D, Ns-Phos	Calcium: 1.2%, phosphate: 1.2%, VD: 0.5 µg/kg 3 times per week	Calcitriol enhanced chondrocyte maturation and restoration of the growth plate architecture; calcitriol increased PTH/PTHrP receptor and d markers of chondrocyte differentiation; daily and intermittent calcitriol had similar effects on endochondral bone growth in phosphorus-loaded rats with renal failure
**Vitamin K and Vitamin D**			
[144]	*n* = 60, female Sprague–Dawley rats after OVX or sham operation; OVX rats were classified into three groups: a VK alone, a VD alone, and combination of VK and VD	VK (menaquinone): 48 mg/100 g diet VD: 0.16 mg/ 100 g diet	VK and VD may have a synergistic effect on reducing bone loss

Abbreviations: VK2, vitamin K2; BMD, bone mineral density; OVX, ovariectomy; TPTD, teriparatide; Gla-OC, γ-carboxylated osteocalcin; VK1, vitamin K1; NTX, 5/6 nephrectomy surgery; vBMD, volumetric bone mineral density; cBMD, cortical bone mineral density; CKD, chronic kidney disease; E2, 17β-estradiol; BW, body weight; Nx, nephrectomized animals; VD, vitamin D; PTH, parathyroid hormone; PTHrP, parathyroid hormone-related protein.

**Table 3:** Vitamin K, vitamin D, and bone health in patients with CKD—clinical evidence.

Reference	Population	Outcome Measure	Main Findings
**Vitamin K**			
[162]	*n* = 468, Adult patients with ESRD referred for single kidney transplant	VK, BMD, parameters of mineral metabolism	Poor vitamin K status is highly prevalent among patients with ESRD and associates with inflammation and low aBMD
[163]	*n* = 20, patients with chronic glomerulonephritis	VK, markers of bone metabolism	MK-4 supplementation suppressed bone loss
[37]	*n* = 141, patients with CKD stages 1–4	PIVKA-II	Subclinical VK deficiency is detectable at just the point in terms of loss of renal function with VC
[160]	*n* = 68, HD patients	VK1, OC, ucOC, iPTH	Suboptimal VK nutriture in HD patients is associated both with increased bone fracture risk and with a high prevalence of hyperparathyroidism
**Vitamin D**			
[164]	*n* = 104, HD patients	VD, transiliac bone biopsy, ALP, iPTH	PTH serum levels are equally elevated in low and high 25D patients; calcitriol levels are constantly low; 25D deficiency resulted in mineralization and bone formation defect; the optimal level of 25D appears to be in the order of 20 to 40 ng/mL
[165]	*n* = 144, HD patients	VD, iPTH, bone densitometry	Increased bone fragility in HD patients is associated with VD deficiency and relative hypoparathyroidism in addition to reduced BMD at the radius
[166]	*n* = 610, elderly women	VD, BMD	Combined calcium and vitamin D_3_ supplementation was effective in reducing the rate of BMD loss in women with moderate CKD
[167]	*n* = 120, patients with stages 1–4 CKD	VD, BMD, OC, NTx, FGF-23	Daily (2000 IU/d) and monthly (40,000 IU/month) VD supplementation for six months in adults with DM and CKD was safe, and it resulted in equivalent adherence and improvements in overall VD status, but only modest changes in markers of bone health
[168]	*n* = 47, CKD patients in stage 3 and 4	PTH, calcium, creatinine, VD	No statistically significant difference between the two treatments: cholecalciferol (4000 IU/d × 1 month, then 2000 IU/d) to doxercalciferol (1 μg/d) in lowering PTH
**Vitamin K and Vitamin D**			
[3]	*n* = 172, patients with stage 3 to 5 CKD	VK, VD, ucOC	Proteinuria was associated with both a suboptimal VD status as well as worse peripheral VK status; high serum ucOC levels were positively associated with phosphate and PTH, and inversely with 25D levels

Abbreviations: ESRD, end-stage renal disease; VK, vitamin K; BMD, bone mineral density; aBMD, areal bone mineral density; MK-4, menaquinone-4; CKD, chronic kidney disease, PIVKA-II, protein induced by VK absence/antagonism II; VC, vascular calcification; HD, hemodialysis; VK1, vitamin K1; OC, osteocalcin; ucOC, uncarboxylated osteocalcin; iPTH, intact parathyroid hormone; ALP, alkaline phosphatase; 25D, 25-hydroxyvitamin D; NTx, N-terminal telopeptide; FGF-23, fibroblast growth factor 23; VD, vitamin D; DM, diabetes mellitus.

## Data Availability

No new data were created or analyzed in this study. Data sharing is not applicable to this article.

## Abbreviations

1,25D	1,25-dihydroxyvitamin D
25D	25-hydroxyvitamin D
ALP	alkaline phosphatase
ApoE4	apolipoprotein E4
BGLAP	bone-Gla-protein; osteocalcin
BMD	bone mineral density
BMP-2	bone morphogenetic protein-2
BMP-7	bone morphogenic protein-7
BW	body weight
CKD	chronic kidney disease
CKD-MBD	chronic kidney disease–mineral bone disorders
CLIA	chemiluminescent immunoassay
cOC	carboxylated osteocalcin
COL1A1	collagen type I
CYP2R1	25-hydroxylase; cytochrome P450 family 2 subfamily R member 1
CYP24A1	cytochrome P450 family 24 subfamily A member 1
CYP27B1	1α-hydroxylase; cytochrome P450 family 27 subfamily B member 1
DCKD	diabetes chronic kidney disease
DECALYOS II	Vitamin D, Calcium, Lyon Study II
DKK1	Dickkopf-related protein 1
dp-ucMGP	desphospho-uncarboxylated matrix Gla protein
DXA	dual X-ray absorptiometry
ECM	extracellular matrix
ELISA	enzyme-linked immunosorbent assay
ESRD	end-stage renal disease
FGF-23	fibroblast growth factor 23
Gas6	growth arrest specific protein 6
GGCX	γ-glutamyl carboxylase
Gla	gamma carboxyglutamic acid
Glu	glutamic acid
GRP	Gla-rich protein
HD	hemodialysis
hMSCs	human bone marrow stromal cells
hOBs	human OBs
hOCs	human OCs
hPBMCs	human peripheral blood mononuclear cells
HPLC	high-performance liquid chromatography
IBSP	integrin-binding sialoprotein
IGFBPs	insulin-like growth factor-binding proteins
IL1*α*	interleukin 1*α*
KDIGO	Kidney Disease Improvement Global Outcomes
KDOQI	Kidney Disease Outcomes Quality Initiative
LC-MS/MS	liquid chromatography-tandem mass spectrometry
LRP5	low-density lipoprotein receptor-related protein 5
m-CSF	colony-stimulating factor
MKs	menaquinones
NFATC1	nuclear factor of activated T cells-c1
NF-*κ*B	nuclear factor kappa-light-chain-enhancer of activated B cells
NR1I2	nuclear receptor subfamily 1 group I member 2
NTx	N-terminal telopeptide
OBs	osteoblasts
OCs	osteoclasts
OPG	osteoprotegerin
OSX	osterix
OVX	ovariectomized
PD	peritoneal dialysis
PGE2	prostaglandin E2
PIVKA-II	protein induced by VK absence/antagonism II
PTH	parathyroid hormone
PXR	pregnane X receptor
RANKL	Receptor Activator for Nuclear Factor κ B Ligand
RIA	radioimmunoassay
RUNX2	Runt-related transcription factor 2
SMAD	small mother against decapentaplegic
SPP1	osteopontin
SXR	steroid and xenobiotic receptor
TM7SF4	transmembrane 7 superfamily member 4
TNFα	tumor necrosis factor α
TRAP	tartrate-resistant acid phosphatase
TRPV6	Transient Receptor Potential Cation Channel Subfamily V Member 6
UBIAD1	UbiA prenyltransferase domain-containing protein
ucOC	uncarboxylated osteocalcin
UV	ultraviolet
VC	vascular calcification
VD	vitamin D
VDBP	VD binding protein
VDR	VD receptor
VEGF	Vascular endothelial growth factor
VK	vitamin K
VK1	vitamin K1
VK2	vitamin K2
VKDPs	VK-dependent proteins
VKOR	VK epoxide reductase
Wnt	Wingless-type
Wnt-10b	Wnt ligand 10b

## References

[1] Banerjee D., Jha V. (2019). Vitamin D and cardiovascular complications of CKD: What’s next?. Clin. J. Am. Soc. Nephrol..

[2] Kidney Disease: Improving Global Outcomes (KDIGO) CKD-MBD Update Work Group (2017). KDIGO 2017 Clinical Practice Guideline Update for the Diagnosis, Evaluation, Prevention, and Treatment of Chronic Kidney Disease-Mineral and Bone Disorder (CKD-MBD). Kidney Int. Suppl..

[3] Holden R.M., Morton A.R., Garland J.S., Pavlov A., Day A.G., Booth S.L. (2010). Vitamins K and D Status in Stages 3–5 Chronic Kidney Disease. Clin. J. Am. Soc. Nephrol..

[4] Garland J.S., Holden R.M., Groome P.A., Lam M., Nolan R.L., Morton A.R., Pickett W. (2008). Prevalence and Associations of Coronary Artery Calcification in Patients With Stages 3 to 5 CKD Without Cardiovascular Disease. Am. J. Kidney Dis..

[5] Florencio-Silva R., Sasso G.R.d.S., Sasso-Cerri E., Simões M.J., Cerri P.S. (2015). Biology of Bone Tissue: Structure, Function, and Factors That Influence Bone Cells. BioMed Res. Int..

[6] Katsimbri P. (2017). The biology of normal bone remodelling. Eur. J. Cancer Care.

[7] Jadoul M., Albert J., Akiba T., Akizawa T., Arab L., Bragggresham J.L., A Mason N., Prutz K.-G., Young E.W., Pisoni R.L. (2006). Incidence and risk factors for hip or other bone fractures among hemodialysis patients in the Dialysis Outcomes and Practice Patterns Study. Kidney Int..

[8] Pimentel A., Ureña-Torres P., Zillikens M.C., Bover J., Cohen-Solal M. (2017). Fractures in patients with CKD—diagnosis, treatment, and prevention: A review by members of the European Calcified Tissue Society and the European Renal Association of Nephrology Dialysis and Transplantation. Kidney Int..

[9] Ureña P., Bernard-Poenaru O., Ostertag A., Baudoin C., Cohen-Solal M., Cantor T., De Vernejoul M.C. (2003). Bone mineral density, biochemical markers and skeletal fractures in haemodialysis patients. Nephrol. Dial. Transplant..

[10] Elliott M.J., Booth S.L., Hopman W.M., Holden R.M. (2014). Assessment of Potential Biomarkers of Subclinical Vitamin K Deficiency in Patients with End-Stage Kidney Disease. Can. J. Kidney Health Dis..

[11] Holden R.M., Iliescu E., Morton A.R., Booth S.L. (2008). Vitamin K Status of Canadian Peritoneal Dialysis Patients. Perit. Dial. Int..

[12] Jean G., Souberbielle J.C., Chazot C. (2017). Vitamin D in Chronic Kidney Disease and Dialysis Patients. Nutrients.

[13] Gois P.H.F., Wolley M., Ranganathan D., Seguro A.C. (2018). Vitamin D Deficiency in Chronic Kidney Disease: Recent Evidence and Controversies. Int. J. Environ. Res. Public Health.

[14] Caravaca-Fontán F., Gonzales-Candia B., Luna E., Caravaca F. (2016). Relative importance of the determinants of serum levels of 25-hydroxy vitamin D in patients with chronic kidney disease. Nefrología.

[15] Melamed M.L., Thadhani R.I. (2011). Vitamin D Therapy in Chronic Kidney Disease and End Stage Renal Disease. Clin. J. Am. Soc. Nephrol..

[16] Thrailkill K.M., Jo C.-H., Cockrell G.E., Moreau C.S., Fowlkes J.L. (2011). Enhanced Excretion of Vitamin D Binding Protein in Type 1 Diabetes: A Role in Vitamin D Deficiency?. J. Clin. Endocrinol. Metab..

[17] Tian X.-Q., Zhao L.-M., Ge J.-P., Zhang Y., Xu Y.-C. (2013). Elevated urinary level of vitamin D-binding protein as a novel biomarker for diabetic nephropathy. Exp. Ther. Med..

[18] Shah N., Bernardini J., Piraino B. (2005). Prevalence and Correction of 25(OH) Vitamin D Deficiency in Peritoneal Dialysis Patients. Perit. Dial. Int..

[19] McCabe K.M., Booth S.L., Fu X., Ward E., Adams M.A., Holden R.M. (2017). Vitamin K Metabolism in a Rat Model of Chronic Kidney Disease. Am. J. Nephrol..

[20] de Oliveira R.B., Stinghen A.E.M., Massy Z.A. (2020). Vitamin K role in mineral and bone disorder of chronic kidney disease. Clin. Chim. Acta.

[21] Holick M.F., Binkley N.C., Bischoff-Ferrari H.A., Gordon C.M., Hanley D.A., Heaney R.P., Murad M.H., Weaver C.M. (2011). Evaluation, Treatment, and Prevention of Vitamin D Deficiency: An Endocrine Society Clinical Practice Guideline. J. Clin. Endocrinol. Metab..

[22] Ennis J.L., Worcester E.M., Coe F.L., Sprague S.M. (2015). Current recommended 25-hydroxyvitamin D targets for chronic kidney disease management may be too low. J. Nephrol..

[23] Cranenburg E.C.M., Schurgers L.J., Uiterwijk H.H., Beulens J.W., Dalmeijer G.W., Westerhuis R., Magdeleyns E.J., Herfs M., Vermeer C., Laverman G.D. (2012). Vitamin K intake and status are low in hemodialysis patients. Kidney Int..

[24] Booth S.L. (2012). Vitamin K: Food composition and dietary intakes. Food Nutr. Res..

[25] Thijssen H.H.W., Drittij-Reijnders M.J. (1994). Vitamin K distribution in rat tissues: Dietary phylloquinone is a source of tissue menaquinone-4. Br. J. Nutr..

[26] Nakagawa K., Hirota Y., Sawada N., Yuge N., Watanabe M., Uchino Y., Okuda N., Shimomura Y., Suhara Y., Okano T. (2010). Identification of UBIAD1 as a novel human menaquinone-4 biosynthetic enzyme. Nat. Cell Biol..

[27] Silaghi C.N., Ilyés T., Filip V.P., Farcaș M., Van Ballegooijen A.J., Crăciun A.M. (2019). Vitamin K Dependent Proteins in Kidney Disease. Int. J. Mol. Sci..

[28] Tabb M.M., Sun A., Zhou C., Grün F., Errandi J., Romero K., Pham H., Inoue S., Mallick S., Lin M. (2003). Vitamin K2 Regulation of Bone Homeostasis Is Mediated by the Steroid and Xenobiotic Receptor SXR. J. Biol. Chem..

[29] Ichikawa T., Horie-Inoue K., Ikeda K., Blumberg B., Inoue S. (2006). Steroid and Xenobiotic Receptor SXR Mediates Vitamin K2-activated Transcription of Extracellular Matrix-related Genes and Collagen Accumulation in Osteoblastic Cells. J. Biol. Chem..

[30] Booth S.L., Pennington J.A., Sadowski J.A. (1996). Food sources and dietary intakes of vitamin K-1 (phylloquinone) in the American diet: Data from the FDA Total Diet Study. J. Am. Diet. Assoc..

[31] Schurgers L.J., Vermeer C. (2000). Determination of phylloquinone and menaquinones in food. Effect of food matrix on circulating vitamin K concentrations. Haemostasis.

[32] Schurgers L.J., Vermeer C. (2002). Differential lipoprotein transport pathways of K-vitamins in healthy subjects. Biochim. Biophys. Acta BBA Gen. Subj..

[33] Akbari S., Rasouli-Ghahroudi A.A. (2018). Vitamin K and Bone Metabolism: A Review of the Latest Evidence in Preclinical Studies. BioMed Res. Int..

[34] Tie J.-K., Stafford D.W. (2016). Structural and functional insights into enzymes of the vitamin K cycle. J. Thromb. Haemost..

[35] Paroni R., Faioni E.M., Razzari C., Fontana G., Cattaneo M. (2009). Determination of vitamin K1 in plasma by solid phase extraction and HPLC with fluorescence detection. J. Chromatogr. B.

[36] Caluwé R., Verbeke F., De Vriese A.S. (2018). Evaluation of vitamin K status and rationale for vitamin K supplementation in dialysis patients. Nephrol. Dial. Transplant..

[37] Voong K., Harrington D., Goldsmith D. (2013). Vitamin K status in chronic kidney disease: A report of a study and a mini-review. Int. Urol. Nephrol..

[38] Iwamoto J., Matsumoto H., Takeda T., Sato Y., Yeh J.K. (2010). Effects of Vitamin K2 on Cortical and Cancellous Bone Mass, Cortical Osteocyte and Lacunar System, and Porosity in Sciatic Neurectomized Rats. Calcif. Tissue Int..

[39] Nagata Y., Inaba M., Imanishi Y., Okazaki H., Yamada S., Mori K., Shoji S., Koyama H., Okuno S. (2015). Increased undercar-boxylated osteocalcin/intact osteocalcin ratio in patients undergoing hemodialysis. Osteoporos. Int..

[40] Fusaro M., Noale M., Viola V., Galli F., Tripepi G., Vajente N., Plebani M., Zaninotto M., Guglielmi G., Miotto D. (2012). Vitamin K, vertebral fractures, vascular calcifications, and mortality: VItamin K Italian (VIKI) dialysis study. J. Bone Miner. Res..

[41] Westenfeld R., Krueger T., Schlieper G., Cranenburg E.C., Magdeleyns E.J., Heidenreich S. (2012). Effect of vitamin K2 supple-mentation on functional vitamin K deficiency in hemodialysis patients: A randomized trial. Am. J. Kidney Dis..

[42] Cozzolino M., Cianciolo G., Podestà M.A., Ciceri P., Galassi A., Gasperoni L., La Manna G. (2020). Current Therapy in CKD Patients Can Affect Vitamin K Status. Nutrients.

[43] Cozzolino M., Mangano M., Galassi A., Ciceri P., Messa P., Nigwekar S. (2019). Vitamin K in Chronic Kidney Disease. Nutrients.

[44] Stankowiak-Kulpa H., Krzyżanowska P., Kozioł L., Grzymisławski M., Wanic-Kossowska M., Moczko J., Walkowiak J. (2011). Vitamin K status in peritoneally dialyzed patients with chronic kidney disease. Acta Biochim. Pol..

[45] Jansz T.T., Neradova A., Van Ballegooijen A.J., Verhaar M.C., Vervloet M.G., Schurgers L.J., Van Jaarsveld B.C. (2018). The role of kidney transplantation and phosphate binder use in vitamin K status. PLoS ONE.

[46] Hauschka P.V., Lian J.B., Cole D.E., Gundberg C.M. (1989). Osteocalcin and matrix Gla protein: Vitamin K-dependent proteins in bone. Physiol. Rev..

[47] Carvallo L., Henriquez B., Paredes R., Olate J., Onate S., Van Wijnen A.J., Lian J.B., Stein G.S., Stein J.L., Montecino M. (2007). 1α,25-dihydroxy vitamin D_3_-enhanced expression of the osteocalcin gene involves increased promoter occupancy of basal transcription regulators and gradual recruitment of the 1α,25-dihydroxy vitamin D_3_ receptor-SRC-1 coactivator complex. J. Cell. Physiol..

[48] Bouleftour W., Bouët G., Granito R.N., Thomas M., Linossier M.-T., Vanden-Bossche A., Aubin J.E., Lafage-Proust M.-H., Vico L., Malaval L. (2014). Blocking the Expression of Both Bone Sialoprotein (BSP) and Osteopontin (OPN) Impairs the Anabolic Action of PTH in Mouse Calvaria Bone. J. Cell. Physiol..

[49] Turner M.E., Adams M.A., Holden R.M. (2018). The Vitamin K Metabolome in Chronic Kidney Disease. Nutrients.

[50] Pilkey R.M., Morton A.R., Boffa M.B., Noordhof C., Day A.G., Su Y., Miller L.M., Koschinsky M.L., Booth S.L. (2007). Subclinical Vitamin K Deficiency in Hemodialysis Patients. Am. J. Kidney Dis..

[51] Verstuyf A., Carmeliet G., Bouillon R., Mathieu C. (2010). Vitamin D: A pleiotropic hormone. Kidney Int..

[52] Levin A., Li Y.C. (2005). Vitamin D and its analogues: Do they protect against cardiovascular disease in patients with kidney disease?. Kidney Int..

[53] DeLuca H.F. (2014). History of the discovery of vitamin D and its active metabolites. BoneKEy Rep..

[54] Holick M., Frommer J., McNeill S., Richtand N., Henley J., Potts J. (1977). Photometabolism of 7-dehydrocholesterol to previtamin D_3_ in skin. Biochem. Biophys. Res. Commun..

[55] Dusso A.S., Brown A.J., Slatopolsky E. (2005). Vitamin D. Am. J. Physiol. Ren. Physiol..

[56] Fraser D.R., Kodicek E. (1970). Unique Biosynthesis by Kidney of a Biologically Active Vitamin D Metabolite. Nat. Cell Biol..

[57] Drechsler C., Pilz S., Obermayer-Pietsch B., Verduijn M., Tomaschitz A., Krane V., Espe K., Dekker F., Brandenburg V.M., März W. (2010). Vitamin D deficiency is associated with sudden cardiac death, combined cardiovascular events, and mortality in haemodialysis patients. Eur. Heart J..

[58] Peterlik M., Cross H.S. (2005). Vitamin D and calcium deficits predispose for multiple chronic diseases. Eur. J. Clin. Investig..

[59] Ramasamy I. (2020). Vitamin D Metabolism and Guidelines for Vitamin D Supplementation. Clin. Biochem. Rev..

[60] Christakos S., Dhawan P., Verstuyf A., Verlinden L., Carmeliet G. (2016). Vitamin D: Metabolism, Molecular Mechanism of Action, and Pleiotropic Effects. Physiol. Rev..

[61] Khundmiri S.J., Murray R.D., Lederer E. (2016). PTH and Vitamin D. Compr. Physiol..

[62] Goltzman D., Mannstadt M., Marcocci C. (2018). Physiology of the Calcium-Parathyroid Hormone-Vitamin D Axis. Front. Horm. Res..

[63] Lombardi G., Ziemann E., Banfi G., Corbetta S. (2020). Physical activity-dependent regulation of parathyroid hormone and calci-um-phosphorous metabolism. Int. J. Mol. Sci..

[64] Jacquillet G., Unwin R.J. (2019). Physiological regulation of phosphate by vitamin D, parathyroid hormone (PTH) and phosphate (Pi). Pflügers Archiv Eur. J. Physiol..

[65] Cardoso M.P., Pereira L.A.L. (2019). Native vitamin D in pre-dialysis chronic kidney disease. Nefrologia.

[66] A Schmidt J. (2006). Measurement of 25-Hydroxyvitamin D Revisited. Clin. Chem..

[67] Terry A.H., Sandrock T., Meikle A.W. (2005). Measurement of 25-Hydroxyvitamin D by the Nichols ADVANTAGE, DiaSorin LIAISON, DiaSorin RIA, and Liquid Chromatography–Tandem Mass Spectrometry. Clin. Chem..

[68] Sirajudeen S., Shah I., Al Menhali A. (2019). A Narrative Role of Vitamin D and Its Receptor: With Current Evidence on the Gastric Tissues. Int. J. Mol. Sci..

[69] Shah I., James B., Barker J., Petroczi A., Naughton D.P. (2011). Misleading measures in Vitamin D analysis: A novel LC-MS/MS assay to account for epimers and isobars. Nutr. J..

[70] Holick M.F. (2007). Vitamin D Deficiency. N. Engl. J. Med..

[71] Mehrotra R., Kermah D., Budoff M., Salusky I.B., Mao S.S., Gao Y.L., Takasu J., Adler S., Norris K. (2008). Hypovitaminosis D in Chronic Kidney Disease. Clin. J. Am. Soc. Nephrol..

[72] Huang Y., Yu H., Lu J., Guo K., Zhang L., Bao Y., Chen H., Jia W. (2012). Oral Supplementation with Cholecalciferol 800 IU Ameliorates Albuminuria in Chinese Type 2 Diabetic Patients with Nephropathy. PLoS ONE.

[73] Capelli I., Cianciolo G., Gasperoni L., Galassi A., Ciceri P., Cozzolino M. (2020). Nutritional vitamin D in CKD: Should we measure? Should we treat?. Clin. Chim. Acta.

[74] Çankaya E., Bilen Y., Keleş M., Uyanık A., Akbaş M., Güngör A., Arslan Ş., Aydınlı B. (2015). Comparison of Serum Vitamin D Levels Among Patients With Chronic Kidney Disease, Patients in Dialysis, and Renal Transplant Patients. Transplant. Proc..

[75] Ravani P., Malberti F., Tripepi G., Pecchini P., Cutrupi S., Pizzini P., Mallamaci F., Zoccali C. (2009). Vitamin D levels and patient outcome in chronic kidney disease. Kidney Int..

[76] Mehrotra R., Kermah D.A., Salusky I.B., Wolf M.S., Thadhani R.I., Chiu Y.-W., Martins D., Adler S.G., Norris K.C. (2009). Chronic kidney disease, hypovitaminosis D, and mortality in the United States. Kidney Int..

[77] KDOQI US Commentary on the 2017 KDIGO Clinical Practice Guideline Update for the Diagnosis, Evaluation, Prevention, and Treatment of Chronic Kidney Disease—Mineral and Bone Disorder (CKD-MBD). https://www.ajkd.org/action/showPdf?pii=S0272-6386%2817%2930898-3.

[78] Koshihara Y., Hoshi K., Okawara R., Ishibashi H., Yamamoto S. (2003). Vitamin K stimulates osteoblastogenesis and inhibits osteoclastogenesis in human bone marrow cell culture. J. Endocrinol..

[79] Li W., Zhang S., Liu J., Liu Y., Liang Q. (2019). Vitamin K2 stimulates Mc3T3‑E1 osteoblast differentiation and mineralization through autophagy induction. Mol. Med. Rep..

[80] Urayama S., Kawakami A., Nakashima T., Tsuboi M., Yamasaki S., Hida A., Ichinose Y., Nakamura H., Ejima E., Aoyagi T. (2000). Effect of vitamin K2 on osteoblast apoptosis: Vitamin K2 inhibits apoptotic cell death of human osteoblasts induced by Fas, proteasome inhibitor, etoposide, and staurosporine. J. Lab. Clin. Med..

[81] Katsuyama H., Saijoh K., Otsuki T., Tomita M., Fukunaga M., Sunami S. (2007). Menaquinone-7 regulates gene expression in osteoblastic MC3T3E1 cells. Int. J. Mol. Med..

[82] Yamaguchi M., Weitzmann M.N. (2011). Vitamin K2 stimulates osteoblastogenesis and suppresses osteoclastogenesis by suppressing NF-κB activation. Int. J. Mol. Med..

[83] Poon C.C., Li R.W., Seto S.W., Kong S.K., Ho H.P., Hoi M.P., Lee S.M., Ngai S.M., Chan S.W., Leung G.P. (2015). In vitro vitamin K2 and 1α,25-dihydroxyvitamin D_3_ combination enhances osteoblasts anabolism of diabetic mice. Eur. J. Pharmacol..

[84] Kameda T., Miyazawa K., Mori Y., Yuasa T., Shiokawa M., Nakamaru Y., Mano H., Hakeda Y., Kameda A., Kumegawa M. (1996). Vitamin K2Inhibits Osteoclastic Bone Resorption by Inducing Osteoclast Apoptosis. Biochem. Biophys. Res. Commun..

[85] Yamaguchi M., Uchiyama S., Tsukamoto Y. (2003). Inhibitory effect of menaquinone-7 (vitamin K2) on the bone-resorbing fac-tors-induced bone resorption in elderly female rat femoral tissues in vitro. Mol. Cell. Biochem..

[86] Pereira R.C., Salusky I.B., Bowen R.E., Freymiller E.G., Wesseling-Perry K. (2019). Vitamin D sterols increase FGF23 expression by stimulating osteoblast and osteocyte maturation in CKD bone. Bone.

[87] Zhou S., Glowacki J., Kim S.W., Hahne J., Geng S., Mueller S.M., Shen L., Bleiberg I., LeBoff M.S. (2012). Clinical characteristics influence in vitro action of 1,25-dihydroxyvitamin D_3_ in human marrow stromal cells. J. Bone Miner. Res..

[88] Al Saedi A., Myers D.E., Stupka N., Duque G. (2020). 1,25(OH)_2_D_3_ ameliorates palmitate-induced lipotoxicity in human primary osteoblasts leading to improved viability and function. Bone.

[89] Allard L., Demoncheaux N., Machuca-Gayet I., Georgess D., Coury-Lucas F., Jurdic P., Bacchetta J. (2015). Biphasic Effects of Vitamin D and FGF23 on Human Osteoclast Biology. Calcif. Tissue Int..

[90] Ji L., Gao J., Kong R., Gao Y., Ji X., Zhao D. (2019). Autophagy exerts pivotal roles in regulatory effects of 1α,25-(OH)_2_D_3_ on the osteoclastogenesis. Biochem. Biophys. Res. Commun..

[91] Zhou S., Leboff M.S., Waikar S.S., Glowacki J. (2013). Vitamin D metabolism and action in human marrow stromal cells: Effects of chronic kidney disease. J. Steroid. Biochem. Mol. Biol..

[92] Pereira R.C., Salusky I.B., Roschger P., Klaushofer K., Yadin O., Freymiller E.G., Bowen R., Delany A.M., Fratzl-Zelman N., Wesseling-Perry K. (2018). Impaired osteocyte maturation in the pathogenesis of renal osteodystrophy. Kidney Int..

[93] Meng F., Bertucci C., Gao Y., Li J., Luu S., LeBoff M.S., Glowacki J., Zhou S. (2020). Fibroblast growth factor 23 counters vitamin D metabolism and action in human mesenchymal stem cells. J. Steroid Biochem. Mol. Biol..

[94] Suda T., Ueno Y., Fujii K., Shinki T. (2002). Vitamin D and bone. J. Cell. Biochem..

[95] Van Leeuwen J.P., Van Driel M., Bemd G.J.V.D., Pols H.A. (2001). Vitamin D control of osteoblast function and bone extracellular matrix mineralization. Crit. Rev. Eukaryot. Gene Expr..

[96] Zarei A., Morovat A., Javaid K., Brown C.P. (2016). Vitamin D receptor expression in human bone tissue and dose-dependent activation in resorbing osteoclasts. Bone Res..

[97] Ženata O., Marcalíková A., Vrzal R. (2019). The effect of caffeine on calcitriol-inducible vitamin D receptor-controlled gene expres-sion in intestinal and osteoblastic cells. Calcif. Tissue Int..

[98] Turner A.G., Hanrath M.A., Morris H.A., Atkins G.J., Anderson P.H. (2014). The local production of 1,25(OH)_2_D_3_ promotes osteoblast and osteocyte maturation. J. Steroid Biochem. Mol. Biol..

[99] Van de Peppel J., van Leeuwen J.P. (2014). Vitamin D and gene networks in human osteoblasts. Front. Physiol..

[100] Meyer M.B., Benkusky N.A., Lee C.-H., Pike J.W. (2014). Genomic Determinants of Gene Regulation by 1,25-Dihydroxyvitamin D_3_ during Osteoblast-lineage Cell Differentiation. J. Biol. Chem..

[101] Geng S., Zhou S., Bi Z., Glowacki J. (2013). Vitamin D metabolism in human bone marrow stromal (mesenchymal stem) cells. Metabolism.

[102] Van Driel M., Koedam M., Buurman C., Hewison M., Chiba H., Uitterlinden A., Pols H., Van Leeuwen J. (2006). Evidence for auto/paracrine actions of vitamin D in bone: 1a-hydroxylase expression and activity in human bone cells. FASEB J..

[103] Duque G., El Abdaimi K., E Henderson J., Lomri A., Kremer R. (2004). Vitamin D inhibits Fas ligand-induced apoptosis in human osteoblasts by regulating components of both the mitochondrial and Fas-related pathways. Bone.

[104] Zhang X., Zanello L.P. (2008). Vitamin D Receptor-Dependent 1α,25(OH)_2_ Vitamin D_3_-Induced Anti-Apoptotic PI3K/AKT Signaling in Osteoblasts. J. Bone Miner. Res..

[105] Weinstein R.S., Manolagas S.C. (2000). Apoptosis and osteoporosis. Am. J. Med..

[106] Prince M., Banerjee C., Javed A., Green J., Lian J.B., Stein G.S., Bodine P.V., Komm B.S. (2001). Expression and regulation of Runx2/Cbfa1 and osteoblast phenotypic markers during the growth and differentiation of human osteoblasts. J. Cell. Biochem..

[107] Maehata Y., Takamizawa S., Ozawa S., Kato Y., Sato S., Kubota E., Hata R.-I. (2006). Both direct and collagen-mediated signals are required for active vitamin D_3_-elicited differentiation of human osteoblastic cells: Roles of osterix, an osteoblast-related transcription factor. Matrix Biol..

[108] Piek E., Sleumer L.S., van Someren E.P., Heuver L., de Haan J.R., de Grijs I., Gilissen C., Hendriks J.M., van Ravestein-van Os R.I., Bauerschmidt S. (2010). Osteo-transcriptomics of human mesenchymal stem cells: Accelerated gene expression and osteoblast differentiation induced by vitamin D reveals c-MYC as an enhancer of BMP2-induced osteo-genesis. Bone.

[109] Kveiborg M., Flyvbjerg A., Eriksen E.F., Kassem M. (2001). 1,25-Dihydroxyvitamin D_3_ stimulates the production of insulin-like growth factor-binding proteins-2, -3 and -4 in human bone marrow stromal cells. Eur. J. Endocrinol..

[110] Zhang H., Li H. (2018). Tricin enhances osteoblastogenesis through the regulation of Wnt/β-catenin signaling in human mesenchymal stem cells. Mech. Dev..

[111] Xiong Y., Zhang Y., Xin N., Yuan Y., Zhang Q., Gong P., Wu Y. (2017). 1α,25-Dihydroxyvitamin D_3_ promotes osteogenesis by promoting Wnt signaling pathway. J. Steroid Biochem. Mol. Biol..

[112] Jo S., Yoon S., Lee S.Y., Kim S.Y., Park H., Han J., Choi S.H., Han J.-S., Yang J.-H., Kim T.-H. (2020). DKK1 Induced by 1,25D3 Is Required for the Mineralization of Osteoblasts. Cells.

[113] Hocking L.J., Whitehouse C., Helfrich M.H. (2012). Autophagy: A new player in skeletal maintenance?. J. Bone Miner. Res..

[114] Nollet M., Santucci-Darmanin S., Breuil V., Al-Sahlanee R., Cros C., Topi M., Momier D., Samson M., Pagnotta S., Cailleteau L. (2014). Autophagy in osteoblasts is involved in mineralization and bone homeostasis. Autophagy.

[115] Mayer H., Bertram H., Lindenmaier W., Korff T., Weber H., Weich H. (2005). Vascular endothelial growth factor (VEGF-A) ex-pression in human mesenchymal stem cells: Autocrine and paracrine role on osteoblastic and endothelial differentiation. J. Cell. Biochem..

[116] Wang D.S., Yamazaki K., Nohtomi K., Shizume K., Ohsumi K., Shibuya M., Demura H., Sato K. (2009). Increase of vascular endothelial growth factor mRNA expression by 1,25-dihydroxyvitamin D_3_ in human osteoblast-like cells. J. Bone Miner. Res..

[117] Neve A., Cantatore F.P., Corrado A., Gaudio A., Ruggieri S., Ribatti D. (2013). In vitro and in vivo angiogenic activity of osteo-arthritic and osteoporotic osteoblasts is modulated by VEGF and vitamin D_3_ treatment. Regul. Pept..

[118] Atkins G.J., Kostakis P., Pan B., Farrugia A., Gronthos S., Evdokiou A., Harrison K., Findlay D.M., Zannettino A.C.W. (2003). RANKL Expression Is Related to the Differentiation State of Human Osteoblasts. J. Bone Miner. Res..

[119] Woeckel V., Alves R., Swagemakers S., Eijken M., Chiba H., Van Der Eerden B., Van Leeuwen J. (2010). 1α,25-(OH)_2_D_3_ acts in the early phase of osteoblast differentiation to enhance mineralization via accelerated production of mature matrix vesicles. J. Cell. Physiol..

[120] Woeckel V., Van Der Eerden B., Schreuders-Koedam M., Eijken M., Van Leeuwen J. (2013). 1α,25-dihydroxyvitamin D_3_ stimulates activin A production to fine-tune osteoblast-induced mineralization. J. Cell. Physiol..

[121] Atkins G.J., Anderson P.H., Findlay D.M., Welldon K.J., Vincent C., Zannettino A.C., O’Loughlin P.D., Morris H.A. (2007). Metabolism of vitamin D_3_ in human osteoblasts: Evidence for autocrine and paracrine activities of 1 alpha,25-dihydroxyvitamin D_3_. Bone.

[122] Li J.J., Sodek J. (1993). Cloning and characterization of the rat bone sialoprotein gene promoter. Biochem. J..

[123] Ormsby R.T., Findlay D.M., Kogawa M., Anderson P.H., Morris H.A., Atkins G.J. (2014). Analysis of vitamin D metabolism gene expression in human bone: Evidence for autocrine control of bone remodelling. J. Steroid Biochem. Mol. Biol..

[124] Giner M., Rios M.J., Montoya M.J., Vázquez M.A., Naji L., Pérez-Cano R. (2009). RANKL/OPG in primary cultures of osteoblasts from post-menopausal women. Differences between osteoporotic hip fractures and osteoarthritis. J. Steroid Biochem. Mol. Biol..

[125] Yasuda H., Shima N., Nakagawa N., Yamaguchi K., Kinosaki M., Mochizuki S.-I., Tomoyasu A., Yano K., Goto M., Murakami A. (1998). Osteoclast differentiation factor is a ligand for osteoprotegerin/osteoclastogenesis-inhibitory factor and is identical to TRANCE/RANKL. Proc. Natl. Acad. Sci. USA.

[126] Bikle D.D. (2012). Vitamin D and bone. Curr. Osteoporos. Rep..

[127] Takahashi N., Udagawa N., Suda T. (2014). Vitamin D endocrine system and osteoclasts. Bonekey Rep..

[128] Suda T., Takahashi N., Abe E. (1992). Role of vitamin D in bone resorption. J. Cell. Biochem..

[129] Kogawa M., Findlay D.M., Anderson P.H., Ormsby R., Vincent C., Morris H.A., Atkins G.J. (2010). Osteoclastic Metabolism of 25(OH)-Vitamin D_3_: A Potential Mechanism for Optimization of Bone Resorption. Endocrinology.

[130] Sakai S., Takaishi H., Matsuzaki K., Kaneko H., Furukawa M., Miyauchi Y., Shiraishi A., Saito K., Tanaka A., Taniguchi T. (2009). 1-Alpha, 25-dihydroxy vitamin D_3_ inhibits osteoclastogenesis through IFN-beta-dependent NFATc1 suppression. J. Bone Miner. Metab..

[131] Kudo O., Sabokbar A., Pocock A., Itonaga I., Athanasou N. (2002). Isolation of Human Osteoclasts Formed In Vitro: Hormonal Effects on the Bone-Resorbing Activity of Human Osteoclasts. Calcif. Tissue Int..

[132] Kim T.-H., Lee B., Kwon E., Choi C.H., Sung I.-H., Kim Y., Sohn J., Ji J.D. (2013). 1,25-dihydroxyvitamin D_3_ inhibits directly human osteoclastogenesis by down-regulation of the c-Fms and RANK expression. Jt. Bone Spine.

[133] Kim H., Baek S., Hong S.M., Lee J., Jung S.M., Lee J., Cho M., Kwok S.K., Park S.H. (2020). 1,25-dihydroxy vitamin D_3_ and interleukin-6 blockade synergistically regulate rheumatoid arthritis by suppressing interleukin-17 production and osteoclas-togenesis. J. Korean Med. Sci..

[134] Bennett C.N., Ouyang H., Ma Y.L., Zeng Q., Gerin I., Sousa K.M., Lane T.F., Krishnan V., Hankenson K.D., MacDougald O.A. (2007). Wnt10b Increases Postnatal Bone Formation by Enhancing Osteoblast Differentiation. J. Bone Miner. Res..

[135] Lu C.-L., Shyu J.-F., Wu C.-C., Hung C.-F., Liao M.-T., Liu W.-C., Zheng C.-M., Hou Y.-C., Lin Y.-F., Lu K.-C. (2018). Association of Anabolic Effect of Calcitriol with Osteoclast-Derived Wnt 10b Secretion. Nutrients.

[136] DeSelm C.J., Miller B.C., Zou W., Beatty W.L., van Meel E., Takahata Y., Klumperman J., Tooze S.A., Teitelbaum S.L., Virgin H.W. (2011). Autophagy Proteins Regulate the Secretory Component of Osteoclastic Bone Resorption. Dev. Cell.

[137] Sul O.J., Park H.J., Son H.J., Choi H.S. (2017). Lipopolysaccharide (LPS)-induced autophagy is responsible for enhanced osteo-clastogenesis. Mol. Cells.

[138] Iwamoto J., Seki A., Sato Y., Matsumoto H. (2011). Vitamin K2 Improves Renal Function and Increases Femoral Bone Strength in Rats with Renal Insufficiency. Calcif. Tissue Int..

[139] Akiyama Y., Hara K., Kobayashi M., Tomiuga T., Nakamura T. (1999). Inhibitory effect of vitamin K2 (menatetrenone) on bone resorption in ovariectomized rats: A histomorphometric and dual energy X-ray absorptiometric study. Jpn. J. Pharmacol..

[140] Nagura N., Komatsu J., Iwase H., Hosoda H., Ohbayashi O., Nagaoka I., Kaneko K. (2015). Effects of the combination of vitamin K and teriparatide on the bone metabolism in ovariectomized rats. Biomed. Rep..

[141] Iwasaki Y., Yamato H., Murayama H., Sato M., Takahashi T., Ezawa I., Kurokawa K., Fukagawa M. (2002). Maintenance of trabecular structure and bone volume by vitamin K 2 in mature rats with long-term tail suspension. J. Bone Miner. Metab..

[142] Kim M., Na W., Sohn C. (2013). Vitamin K1 (phylloquinone) and K2 (menaquinone-4) supplementation improves bone formation in a high-fat diet-induced obese mice. J. Clin. Biochem. Nutrients.

[143] Iwasaki Y., Yamato H., Murayama H., Sato M., Takahashi T., Ezawa I., Kurokawa K., Fukagawa M. (2003). Combination use of vitamin K 2 further increases bone volume and ameliorates extremely low turnover bone induced by bisphosphonate therapy in tail-suspension rats. J. Bone Miner. Metab..

[144] Matsunaga S., Ito H., Sakou T. (1999). The effect of vitamin K and D supplementation on ovariectomy-induced bone loss. Calcif. Tissue Int..

[145] Otomo H., Sakai A., Ikeda S., Tanaka S., Ito M., Phipps R.J., Nakamura T. (2004). Regulation of mineral-to-matrix ratio of lumbar trabecular bone in ovariectomized rats treated with risedronate in combination with or without vitamin K2. J. Bone Miner. Metab..

[146] Matsumoto Y., Mikuni-Takagaki Y., Kozai Y., Miyagawa K., Naruse K., Wakao H., Kawamata R., Kashima I., Sakurai T. (2009). Prior treatment with vitamin K2 significantly improves the efficacy of risedronate. Osteoporos. Int..

[147] Jokihaara J., Pörsti I., Pajamäki I., Vuohelainen T., Jolma P., Kööbi P., Kalliovalkama J., Niemela O., Kannus P., Sievanen H. (2006). Paricalcitol [19-Nor-1,25-(OH)2D2] in the Treatment of Experimental Renal Bone Disease. J. Bone Miner. Res..

[148] Díaz M.N., Rodríguez A.R., Fernandez-Martin J.L., Arias M.S., Rodríguez P.M., Cannata-Andía J.B. (2007). Effects of estradiol, calcitriol and both treatments combined on bone histomorphometry in rats with chronic kidney disease and ovariectomy. Bone.

[149] Sanchez C.P., He Y.Z. (2007). Bone growth during daily or intermittent calcitriol treatment during renal failure with advanced secondary hyperparathyroidism. Kidney Int..

[150] Rutherford W.E., Bordier P., Marie P., Hruska K., Harter H., Greenwalt A., Blondin J., Haddad J., Bricker N., Slatopolsky E. (1977). Phosphate Control and 25-Hydroxycholecalciferol Administration in Preventing Experimental Renal Osteodystrophy in the Dog. J. Clin. Investig..

[151] Jablonski G., Mortensen B.M., Klem K.H., Mosekilde L., Danielsen C.C., Gordeladze J.O. (1995). Vitamin D_3_ analogs and salmon calcitonin partially reverse the development of renal osteodystrophy in rats. Calcif. Tissue Int..

[152] Newman C.L., Tian N., Hammond M.A., Wallace J.M., Brown D.M., Chen N.X., Moe S.M., Allen M.R. (2016). Calcitriol sup-pression of parathyroid hormone fails to improve skeletal properties in an animal model of chronic kidney disease. Am. J. Nephrol..

[153] De Schutter T.M., Behets G.J., Jung S., Neven E., D’Haese P.C., Querfeld U. (2012). Restoration of Bone Mineralization by Cinacalcet is Associated with a Significant Reduction in Calcitriol-Induced Vascular Calcification in Uremic Rats. Calcif. Tissue Int..

[154] Bisson S.-K., Ung R.-V., Picard S., Valade D., Agharazii M., Larivière R., Mac-Way F. (2018). High calcium, phosphate and calcitriol supplementation leads to an osteocyte-like phenotype in calcified vessels and bone mineralisation defect in uremic rats. J. Bone Miner. Metab..

[155] Idelevich A., Kerschnitzki M., Shahar R., Monsonego-Ornan E. (2011). 1,25(OH)_2_D_3_ Alters Growth Plate Maturation and Bone Architecture in Young Rats with Normal Renal Function. PLoS ONE.

[156] Wronski T., Halloran B.P., Bikle D.D., Globus R.K., Morey-Holton E.R. (1986). Chronic administration of 1,25-dihydroxyvitamin D_3_: Increased bone but impaired mineralization. Endocrinology.

[157] Lieben L., Masuyama R., Torrekens S., Van Looveren R., Schrooten J., Baatsen P., Lafage-Proust M.-H., Dresselaers T., Feng J.Q., Bonewald L.F. (2012). Normocalcemia is maintained in mice under conditions of calcium malabsorption by vitamin D–induced inhibition of bone mineralization. J. Clin. Investig..

[158] Xue Y., Karaplis A.C., Hendy G.N., Goltzman D., Miao D. (2006). Exogenous 1,25-Dihydroxyvitamin D_3_ Exerts a Skeletal Anabolic Effect and Improves Mineral Ion Homeostasis in Mice that Are Homozygous for Both the 1α-Hydroxylase and Parathyroid Hormone Null Alleles. Endocrinology.

[159] Panda D.K., Miao D., Bolivar I., Li J., Huo R., Hendy G.N., Goltzman D. (2004). Inactivation of the 25-Hydroxyvitamin D 1α-Hydroxylase and Vitamin D Receptor Demonstrates Independent and Interdependent Effects of Calcium and Vitamin D on Skeletal and Mineral Homeostasis. J. Biol. Chem..

[160] Kohlmeier M., Saupe J., Shearer M.J., Schaefer K., Asmus G. (1997). Bone health of adult hemodialysis patients is related to vitamin K status. Kidney Int..

[161] Fusaro M., Tripepi G., Noale M., Plebani M., Zaninotto M., Piccoli A., Naso A., Miozzo D., Giannini S., Avolio M. (2015). Prevalence of Vertebral Fractures, Vascular Calcifications, and Mortality in Warfarin Treated Hemodialysis Patients. Curr. Vasc. Pharmacol..

[162] Evenepoel P., Claes K., Meijers B., Laurent M., Bammens B., Naesens M., Sprangers B., Pottel H., Cavalier E., Kuypers D. (2019). Poor Vitamin K Status Is Associated With Low Bone Mineral Density and Increased Fracture Risk in End-Stage Renal Disease. J. Bone Miner. Res..

[163] Sasaki N., Kusano E., Takahashi H., Ando Y., Yano K., Tsuda E., Asano Y. (2005). Vitamin K2 inhibits glucocorticoid-induced bone loss partly by preventing the reduction of osteoprotegerin (OPG). J. Bone Miner. Metab..

[164] Coen G., Mantella D., Manni M., Balducci A., Nofroni I., Sardella D., Ballanti P., Bonucci E. (2005). 25-hydroxyvitamin D levels and bone histomorphometry in hemodialysis renal osteodystrophy. Kidney Int..

[165] Ambrus C., Almasi C., Berta K., Deák G., Marton A., Molnár M.Z., Németh Z., Horváth C., Lakatos P., Szathmári M. (2010). Vitamin D insufficiency and bone fractures in patients on maintenance hemodialysis. Int. Urol. Nephrol..

[166] Bosworth C., De Boer I.H., Targher G., Kendrick J., Smits G., Chonchol M. (2012). The effect of combined calcium and cholecalciferol supplementation on bone mineral density in elderly women with moderate chronic kidney disease. Clin. Nephrol..

[167] Mager D.R., Jackson S.T., Hoffmann M.R., Jindal K., Senior P.A. (2017). Vitamin D 3 supplementation, bone health and quality of life in adults with diabetes and chronic kidney disease: Results of an open label randomized clinical trial. Clin. Nutr..

[168] Moe S.M., Saifullah A., LaClair R.E., Usman S.A., Yu Z. (2010). A Randomized Trial of Cholecalciferol versus Doxercalciferol for Lowering Parathyroid Hormone in Chronic Kidney Disease. Clin. J. Am. Soc. Nephrol..

[169] Caluwe R., Vandecasteele S., Van Vlem B., Vermeer C., De Vriese A.S. (2014). Vitamin K2 supplementation in haemodialysis patients: A randomized dose-finding study. Nephrol. Dial. Transplant..

[170] Aoun M., Makki M., Azar H., Matta H., Chelala D.N. (2017). High Dephosphorylated-Uncarboxylated MGP in Hemodialysis patients: Risk factors and response to vitamin K2, A pre-post intervention clinical trial. BMC Nephrol..

[171] ClinicalTrials.gov. https://clinicaltrials.gov/ct2/show/record/NCT02976246.

[172] Krueger T., Schlieper G., Schurgers L., Cornelis T., Cozzolino M., Jacobi J., Jadoul M., Ketteler M., Rump L.C., Stenvinkel P. (2014). Vitamin K1 to slow vascular calcification in haemodialysis patients (VitaVasK trial): A rationale and study protocol. Nephrol. Dial. Transplant..

[173] Haroon S.W., Tai B.C., Ling L.H., Teo L., Davenport A., Schurgers L., Teo B.W., Khatri P., Ong C.C., Low S. (2020). Treatment to reduce vascular calcification in he-modialysis patients using vitamin K (Trevasc-HDK): A study protocol for a randomized controlled trial. Medicine.

[174] Oikonomaki T., Papasotiriou M., Ntrinias T., Kalogeropoulou C., Zabakis P., Kalavrizioti D., Papadakis I., Goumenos D.S., Papachristou E. (2019). The effect of vitamin K2 supplementation on vascular calcification in haemodialysis patients: A 1-year follow-up randomized trial. Int. Urol Nephrol..

[175] ClinicalTrials.gov. https://clinicaltrials.gov/ct2/show/NCT02324686.

[176] ClinicalTrials.gov. https://clinicaltrials.gov/ct2/show/NCT02278692.

[177] ClinicalTrials.gov. https://clinicaltrials.gov/ct2/show/NCT04477811.

[178] Levin A., Bakris G., Molitch M., Smulders M., Tian J., Williams L., Andress D. (2007). Prevalence of abnormal serum vitamin D, PTH, calcium, and phosphorus in patients with chronic kidney disease: Results of the study to evaluate early kidney disease. Kidney Int..

[179] Taal M.W., Thurston V., McIntyre N.J., Fluck R.J., McIntyre C.W. (2014). The impact of vitamin D status on the relative increase in fibroblast growth factor 23 and parathyroid hormone in chronic kidney disease. Kidney Int..

[180] Westerberg P.-A., Linde T., Wikström B., Ljunggren Ö., Stridsberg M., Larsson T.E. (2007). Regulation of fibroblast growth factor-23 in chronic kidney disease. Nephrol. Dial. Transplant..

[181] Shardlow A., McIntyre N.J., Fluck R.J., McIntyre C.W., Taal M.W. (2017). Associations of fibroblast growth factor 23, vitamin D and parathyroid hormone with 5-year outcomes in a prospective primary care cohort of people with chronic kidney disease stage 3. BMJ Open.

[182] Metzger M., Houillier P., Gauci C., Haymann J.P., Flamant M., Thervet E., Boffa J.-J., Vrtovsnik F., Froissart M., Stengel B. (2013). Relation Between Circulating Levels of 25(OH) Vitamin D and Parathyroid Hormone in Chronic Kidney Disease: Quest for a Threshold. J. Clin. Endocrinol. Metab..

[183] González E.A., Sachdeva A., Oliver D.A., Martin K.J. (2004). Vitamin D Insufficiency and Deficiency in Chronic Kidney Disease. Am. J. Nephrol..

[184] Ishimura E., Tsuchida T. (2004). Vitamin D deficiency/insufficiency in patients with chronic kidney disease stage 3 and 4—Current concept and its therapeutic strategy. Clin. Calcium.

[185] LaClair R.E., Hellman R.N., Karp S.L., Kraus M., Ofner S., Li Q., Graves K.L., Moe S.M. (2005). Prevalence of Calcidiol Deficiency in CKD: A Cross-Sectional Study Across Latitudes in the United States. Am. J. Kidney Dis..

[186] Memon S., Alam A., Iftikhar S. (2020). Frequency of vitamin D deficiency in chronic kidney disease and its relation with baseline mineral bone markers. J. Pak. Med. Assoc..

[187] Mucsi I., Almási C., Deák G., Marton A., Ambrus C., Berta K., Lakatos P., Szabó A., Horváth C. (2005). Serum 25(OH)-vitamin D levels and bone metabolism in patients on maintenance hemodialysis. Clin. Nephrol..

[188] Ghazali A., Fardellone P., Pruna A., Atik A., Achard J.M., Oprisiu R., Brazier M., Remond A., Morinière P., Garabedian M. (1999). Is low plasma 25-(OH)vitamin D a major risk factor for hyperparathyroidism and Looser’s zones independent of calcitriol?. Kidney Int..

[189] Elder G.J., Mackun K. (2006). 25-Hydroxyvitamin D Deficiency and Diabetes Predict Reduced BMD in Patients with Chronic Kidney Disease. J. Bone Miner. Res..

[190] Lee Y.H., Kim J.E., Roh Y.H., Choi H.R., Rhee Y., Kang D.R., Lim S.K. (2014). The combination of vitamin D deficiency and mild to moderate chronic kidney disease is associated with low bone mineral density and deteriorated femoral microarchitecture: Results from the KNHANES 2008–2011. J. Clin. Endocrinol. Metab..

[191] Brunerová L., Ronová P., Verešová J., Beranová P., Potoèková J., Kasalický P., Rychlík I. (2016). Osteoporosis and impaired trabecular bone score in hemodialysis patients. Kidney Blood Press. Res..

[192] Chan A.M., Johnson D. Vitamin D Therapy (Supplementation) in Early Chronic Kidney Disease. The CARI Guidelines. http://citeseerx.ist.psu.edu/viewdoc/download?doi=10.1.1.363.4591&rep=rep1&type=pdf.

[193] Isakova T., Nickolas T.L., Denburg M., Yarlagadda S., Weiner D.E., Gutiérrez O.M., Bansal V., Rosas S.E., Nigwekar S., Yee J. (2017). KDOQI US Commentary on the 2017 KDIGO Clinical Practice Guideline Update for the Diagnosis, Evaluation, Prevention, and Treatment of Chronic Kidney Disease–Mineral and Bone Disorder (CKD-MBD). Am. J. Kidney Dis..

[194] Cozzolino M., Ciceri P., Galassi A., Mangano M., Carugo S., Capelli I., Cianciolo G. (2019). The Key Role of Phosphate on Vascular Calcification. Toxins.

[195] Mizobuchi M., Ogata H., Koiwa F., Kinugasa E., Akizawa T. (2009). Vitamin D and vascular calcification in chronic kidney disease. Bone.

[196] Vervloet M., Cozzolino M. (2017). Vascular calcification in chronic kidney disease: Different bricks in the wall?. Kidney Int..

[197] Barreto D.V., Barreto F.C., Liabeuf S., Temmar M., Boitte F., Choukroun G., Fournier A., Massy Z.A. (2009). Vitamin D Affects Survival Independently of Vascular Calcification in Chronic Kidney Disease. Clin. J. Am. Soc. Nephrol..

[198] Oksa A., Spustová V., Krivosíková Z., Gazdíková K., Fedelesová V., Lajdová I., Stefíková K., Bernasovská G., Zilinská Z., Dzúrik R. (2008). Effects of long-term cholecalciferol supplementation on mineral metabolism and calciotropic hormones in chronic kidney disease. Kidney Blood Press. Res..

[199] Marckmann P., Agerskov H., Thineshkumar S., Bladbjerg E.-M., Sidelmann J.J., Jespersen J., Nybo M., Rasmussen L.M., Hansen D., Scholze A. (2012). Randomized controlled trial of cholecalciferol supplementation in chronic kidney disease patients with hypovitaminosis D. Nephrol. Dial. Transplant..

[200] A Alvarez J., Law J., E Coakley K., Zughaier S.M., Hao L., Salles K.S., Wasse H., Gutiérrez O.M., Ziegler T.R., Tangpricha V. (2012). High-dose cholecalciferol reduces parathyroid hormone in patients with early chronic kidney disease: A pilot, randomized, double-blind, placebo-controlled trial. Am. J. Clin. Nutr..

[201] Yadav A.K., Kumar V., Kumar V., Banerjee D., Gupta K.L., Jha V. (2017). The Effect of Vitamin D Supplementation on Bone Metabolic Markers in Chronic Kidney Disease. J. Bone Miner. Res..

[202] Chandra P., Binongo J.N.G., Ziegler T.R., Schlanger L.E., Wang W., Someren J.T., Tangpricha V. (2008). Cholecalciferol (Vitamin D_3_) Therapy and Vitamin D Insufficiency in Patients with Chronic Kidney Disease: A Randomized Controlled Pilot Study. Endocr. Pr..

[203] Rucker D., Tonelli M., Coles M.G., Yoo S., Young K., McMahon A.W. (2009). Vitamin D insufficiency and treatment with oral vitamin D_3_ in northern-dwelling patients with chronic kidney disease. J. Nephrol..

[204] Zelnick L.R., de Boer I.H., Kestenbaum B.R., Chonchol M., Kendrick J. (2018). Comparative effects of cholecalciferol and calcitriol on circulating markers of CKD mineral bone disorder: A randomized clinical trial. Clin. J. Am. Soc. Nephrol..

[205] Zisman A.L., Hristova M., Ho L.T., Sprague S.M. (2007). Impact of Ergocalciferol Treatment of Vitamin D Deficiency on Serum Parathyroid Hormone Concentrations in Chronic Kidney Disease. Am. J. Nephrol..

[206] Porter A., Gilmartin C., Srisakul U., Arruda J., Akkina S. (2013). Prevalence of 25-OH Vitamin D Deficiency in a Population of Hemodialysis Patients and Efficacy of an Oral Ergocalciferol Supplementation Regimen. Am. J. Nephrol..

[207] Deville J., Thorp M.L., Tobin L., Gray E., Johnson E.S., Smith D.H. (2006). Effect of ergocalciferol supplementation on serum parathyroid hormone and serum 25-hydroxyvitamin D in chronic kidney disease. Nephrology.

[208] Al-Aly Z., Qazi R.A., González E.A., Zeringue A., Martin K.J. (2007). Changes in Serum 25-Hydroxyvitamin D and Plasma Intact PTH Levels Following Treatment With Ergocalciferol in Patients With CKD. Am. J. Kidney Dis..

[209] Gravesen E., Hofman-Bang J., Lewin E., Olgaard K. (2013). Ergocalciferol treatment and aspects of mineral homeostasis in patients with chronic kidney disease stage 4–5. Scand. J. Clin. Lab. Investig..

[210] Del Valle E., Negri A.L., Fradinger E., Canalis M., Bevione P., Curcelegui M., Bravo M., Puddu M., Marini A., Ryba J. (2014). Weekly high-dose ergocalciferol to correct vitamin D deficiency/insufficiency in hemodialysis patients: A pilot trial. Hemodial. Int..

[211] Wetmore J.B., Kimber C., Mahnken J.D., Stubbs J.R. (2016). Cholecalciferol v. ergocalciferol for 25-hydroxyvitamin D (25(OH)D) repletion in chronic kidney disease: A randomised clinical trial. Br. J. Nutr..

[212] Mangoo-Karim R., Abreu J.D.S., Yanev G.P., Perez N.N., Stubbs J.R., Wetmore J.B. (2015). Ergocalciferol versus Cholecalciferol for Nutritional Vitamin D Replacement in CKD. Nephron.

[213] Glendenning P., Chew G.T., Inderjeeth C.A., Taranto M., Fraser W.D. (2013). Calculated free and bioavailable vitamin D metabolite concentrations in vitamin D-deficient hip fracture patients after supplementation with cholecalciferol and ergocalciferol. Bone.

[214] Iwamoto J., Takeda T., Ichimura S. (2000). Effect of combined administration of vitamin D_3_ and vitamin K2 on bone mineral density of the lumbar spine in postmenopausal women with osteoporosis. J. Orthop. Sci..

[215] Torbergsen A.C., Watne L.O., Wyller T.B., Frihagen F., Strømsøe K., Bøhmer T., Mowe M. (2015). Vitamin PK and 25(OH)D are independently and synergistically associated with a risk for hip fracture in an elderly population: A case control study. Clin. Nutr..

[216] Je S.H., Joo N.-S., Choi B.-H., Kim K.-M., Kim B.-T., Park S.-B., Cho D.-Y., Kim K.-N., Lee D.-J. (2011). Vitamin K Supplement Along with Vitamin D and Calcium Reduced Serum Concentration of Undercarboxylated Osteocalcin While Increasing Bone Mineral Density in Korean Postmenopausal Women over Sixty-Years-Old. J. Korean Med Sci..

[217] Ushiroyama T., Ikeda A., Ueki M. (2002). Effect of continuous combined therapy with vitamin K2 and vitamin D_3_ on bone mineral density and coagulofibrinolysis function in postmenopausal women. Maturitas.

[218] Szulc P., Chapuy M.-C., Meunier P., Delmas P. (1996). Serum undercarboxylated osteocalcin is a marker of the risk of hip fracture: A three year follow-up study. Bone.

[219] Lian J., Stewart C., Puchacz E., Mackowiak S., Shalhoub V., Collart D., Zambetti G., Stein G. (1989). Structure of the rat osteocalcin gene and regulation of vitamin D-dependent expression. Proc. Natl. Acad. Sci. USA.

[220] Miyake N., Hoshi K., Sano Y., Kikuchi K., Tadano K., Koshihara Y. (2001). 1,25-Dihydroxyvitamin D 3 Promotes Vitamin K 2 Metabolism in Human Osteoblasts. Osteoporos. Int..

[221] Furie B.C., Bouchard B.A. (1999). Vitamin K-dependent biosynthesis of gamma-carboxyglutamic acid. Blood.

[222] Price P.A., Baukol S.A. (1981). 1,25-Dihydroxyvitamin D_3_ increases serum levels of the vitamin K-dependent bone protein. Biochem. Biophys. Res. Commun..

[223] Szulc P., Delmasm P.D. (1996). Influence of vitamin D and retinoids on the gammacarboxylation of osteocalcin in human osteosar-coma MG63 cells. Bone.

[224] Goltzman D. (2018). Functions of vitamin D in bone. Histochem. Cell Biol..

[225] Koshihara Y., Hoshi K., Ishibashi H., Shiraki M. (1996). Vitamin K2 promotes 1α,25(OH)_2_ vitamin D_3_-induced mineralization in human periosteal osteoblasts. Calcif. Tissue Int..

[226] Buranasinsup S., Bunyaratavej N. (2015). The Intriguing Correlation between Undercarboxylated Osteocalcin and Vitamin D. J. Med Assoc. Thail..

[227] Van Ballegooijen A.J., Beulens J.W.J., Schurgers L.J., De Koning E.J., Lips P., Van Schoor N.M., Vervloet M.G. (2019). Effect of 6-Month Vitamin D Supplementation on Plasma Matrix Gla Protein in Older Adults. Nutrients.

[228] Clowes J.A., Riggs B.L., Khosla S. (2005). The role of the immune system in the pathophysiology of osteoporosis. Immunol. Rev..

[229] Reddi K., Henderson B., Meghji S., Wilson M., Poole S., Hopper C., Harris M., Hodges S.J. (1995). Interleukin 6 production by lipopolysaccharide-stimulated human fibroblasts is potently inhibited by naphthoquinone (vitamin K) compounds. Interleukin 6 production by lipopolysaccharide-stimulated human fibroblasts is potently inhibited by naphthoquinone (vitamin K) com-pounds. Cytokine.

[230] Koshihara Y., Hoshi K., Shiraki M. (1993). Vitamin K2 (menatetrenone) inhibits prostaglandin synthesis in cultured human osteoblast-like periosteal cells by inhibiting prostaglandin H synthase activity. Biochem. Pharmacol..

[231] Ohsaki Y., Shirakawa H., Hiwatashi K., Furukawa Y., Mizutani T., Komai M. (2006). Vitamin K suppresses lipopolysaccha-ride-induced inflammation in the rat. Biosci. Biotechnol. Biochem..

[232] Shea M.K., Booth S.L., Massaro J.M., Jacques P.F., D’Agostino R.B., Dawson-Hughes B., Ordovas J.M., O’Donnell C.J., Kathiresan S., Keaney J.F. (2008). Vitamin K and vitamin D status: Associations with inflammatory markers in the Framingham Offspring Study. Am. J. Epidemiol..

[233] van Etten E., Mathieu C. (2005). Immunoregulation by 1,25-dihydroxyvitamin D_3_: Basic concepts. J. Steroid. Biochem. Mol. Biol..

[234] Inanir A., Özoran K., Tutkak H., Mermerci B. (2004). The Effects of Calcitriol Therapy on Serum Interleukin-1, Interleukin-6 and Tumour Necrosis Factor-α Concentrations in Post-menopausal Patients with Osteoporosis. J. Int. Med Res..

[235] Zhou C., Assem M., Tay J.C., Watkins P.B., Blumberg B., Schuetz E.G., Thummel K.E. (2006). Steroid and xenobiotic receptor and vitamin D receptor crosstalk mediates CYP24 expression and drug-induced osteomalacia. J. Clin. Investig..

[236] Hosseinpour F., Ellfolk M., Norlin M., Wikvall K. (2007). Phenobarbital suppresses vitamin D_3_ 25-hydroxylase expression: A po-tential new mechanism for drug-induced osteomalacia. Biochem. Biophys. Res. Commun..

[237] Hara K., Akiyama Y., Tomiuga T., Kobayashi M., Nakamura T., Tajima T. (1994). Influence of vitamin D_3_ on inhibitory effect of vitamin K2 on bone loss in ovariectomized rats. Folia Pharmacol. Jpn..

[238] Shiraishi A., Higashi S., Masaki T., Saito M., Ito M., Ikeda S., Nakamura T. (2002). A Comparison of Alfacalcidol and Menatetrenone for the Treatment of Bone Loss in an Ovariectomized Rat Model of Osteoporosis. Calcif. Tissue Int..

[239] Omelka R., Martiniakova M., Svik K., Slovak L., Payer J., Oppenbergerova I., Kovacova V., Babikova M., Soltesova-Prnova M. (2020). The effects of eggshell calcium (Biomin H^®^) and its combinations with alfacalcidol (1α-hydroxyvitamin D_3_) and menaquinone-7 (vitamin K2) on ovariectomy-induced bone loss in a rat model of osteoporosis. J. Anim. Physiol. Anim. Nutr..

[240] Iwamoto J., Yeh J.K., Takeda T., Ichimura S., Sato Y. (2003). Comparative effects of vitamin K and vitamin D supplementation on prevention of osteopenia in calcium-deficient young rats. Bone.

[241] Kuang X., Liu C., Guo X.-F., Li K., Deng Q., Li D. (2020). The combination effect of vitamin K and vitamin D on human bone quality: A meta-analysis of randomized controlled trials. Food Funct..

